# DNA-mediated dimerization on a compact sequence signature controls enhancer engagement and regulation by FOXA1

**DOI:** 10.1093/nar/gky259

**Published:** 2018-04-14

**Authors:** Xuecong Wang, Yogesh Srivastava, Aleksander Jankowski, Vikas Malik, Yuanjie Wei, Ricardo CH del Rosario, Vlad Cojocaru, Shyam Prabhakar, Ralf Jauch

**Affiliations:** 1 CAS Key Laboratory of Regenerative Biology, Joint School of Life Sciences, Guangzhou Institutes of Biomedicine and Health, Chinese Academy of Sciences and Guangzhou Medical University, Guangzhou 511436, China; 2 Genome Regulation Laboratory, Guangdong Provincial Key Laboratory of Stem Cell and Regenerative Medicine, Guangzhou Institutes of Biomedicine and Health, Chinese Academy of Sciences, Guangzhou 510530, China; 3 University of Chinese Academy of Sciences, Beijing 100049, China; 4 Computational and Systems Biology, Genome Institute of Singapore, Singapore 138672, Singapore; 5 Faculty of Mathematics, Informatics and Mechanics, University of Warsaw, 02-097 Warszawa, Poland; 6 Genome Biology Unit, European Molecular Biology Laboratory, 69117 Heidelberg, Germany; 7 Stanley Center for Psychiatric Research, Broad Institute of MIT and Harvard, 75 Ames St., Cambridge MA 02142, USA; 8 Computational Structural Biology Laboratory, Department of Cell and Developmental Biology, Max Planck Institute for Molecular Biomedicine, Röntgenstrasse 20, Münster 48149, Germany; 9 Center for Multiscale Theory and Computation, Westfälische Wilhelms University, 48149 Münster, Germany; 10 School of Biomedical Sciences, Li Ka Shing Faculty of Medicine, The University of Hong Kong, Hong Kong SAR, China

## Abstract

FOXA1 is a transcription factor capable to bind silenced chromatin to direct context-dependent cell fate conversion. Here, we demonstrate that a compact palindromic DNA element (termed ‘DIV’ for its diverging half-sites) induces the homodimerization of FOXA1 with strongly positive cooperativity. Alternative structural models are consistent with either an indirect DNA-mediated cooperativity or a direct protein-protein interaction. The cooperative homodimer formation is strictly constrained by precise half-site spacing. Re-analysis of chromatin immunoprecipitation sequencing data indicates that the DIV is effectively targeted by FOXA1 in the context of chromatin. Reporter assays show that FOXA1-dependent transcriptional activity declines when homodimeric binding is disrupted. In response to phosphatidylinositol-3 kinase inhibition DIV sites pre-bound by FOXA1 such as at the *PVT1/MYC* locus exhibit a strong increase in accessibility suggesting a role of the DIV configuration in the chromatin closed-open dynamics. Moreover, several disease-associated single nucleotide polymorphisms map to DIV elements and show allelic differences in FOXA1 homodimerization, reporter gene expression and are annotated as quantitative trait loci. This includes the rs541455835 variant at the *MAPT* locus encoding the Tau protein associated with Parkinson's disease. Collectively, the DIV guides chromatin engagement and regulation by FOXA1 and its perturbation could be linked to disease etiologies.

## INTRODUCTION

The mechanism by which transcription factor (TF) proteins scan the genome to arrive at functional target sites and to direct changes in chromatin architecture and gene expression programs that confer cellular identities is only poorly understood. Puzzlingly, individual TFs bind only a small subset of their high-affinity consensus sites encoded in the genome. Dimeric TF partnerships can direct site selections and increase specificity by favoring some genomic locations over others. The *forkhead* box A1 (FOXA1) protein belongs to a class of TFs capable to engage their cognate binding sites even in the context of compacted chromatin, which is not accessible to most other TFs (1–4). This way, FOXA1 does so-called ‘pioneering’ work necessary to direct cellular differentiation during organ development (reviewed in (5)), during cell fate programming *in vitro* (6,7) as well as during pathological reprogramming events such as oncogenic transformation (8,9). In mammals, FOXA1 directs liver development and metabolism (10–12), and has important functions in other endodermally derived organs (reviewed in (13)). In breast and prostate epithelia, FOXA1 recruits and collaborates with nuclear hormone receptors, especially estrogen receptor (ER) (14–16) and androgen receptor (AR) by enabling them to access sites they could otherwise not target (17,18). FOXA1 has so far been thought to bind chromatin either as monomer or as heterodimer with nuclear receptors. Whilst it is clear that FOXA1 plays fundamental roles in oncogenesis and development, the mechanism of how FOXA1 engages chromatin to orchestrate regulatory programs and the sequence-function relationships enabling its pioneering function are not resolved (19,20).

FOXA1 possesses an ∼100 amino acid *forkhead* box DNA binding domain (DBD) named after the homologous *Drosophila* gene *fkh* (*forkhead*) (11,21). The *forkhead* DBD was likened to a butterfly with a core consisting of three α-helices and two extended ‘wings’ forming a ‘winged helix’ structure (22). Interestingly, this structure resembles the fold of the linker histones H1/H5, whose role is to compact nucleosomes into higher order structures by binding to the dyad axis of the histone octamer (22,23). The chromatin loosening activity of FOXA1 has been attributed to this similarity and it was suggested that FOXA1 de-compacts nucleosomal arrays by directly competing with linker histones H1 (1). A direct interaction of a C-terminal sequence motif of FOXA1 with the nucleosome core particle was also found to be important for this activity (1). Yet, as a number of structurally unrelated TFs were also reported to be able to bind nucleosomes *in vitro*, the similarity to H1 does not appear to be an essential feature of pioneer TFs (24).

**Figure 1.**
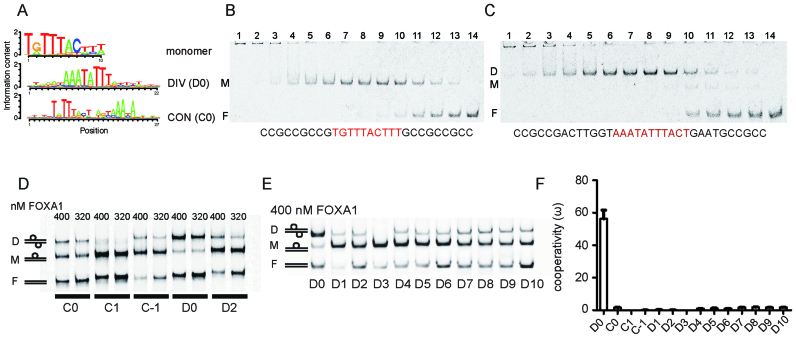
FOXA1 cooperates on the DIV motif with strict constraints on half-site spacing. (**A**) Sequence logos representing position weight matrices (PWMs) of the FOXA1 monomer motif as well as logos for the composite DIV (D0) and CON (C0) motifs. The source PWMs are in Supplementary Table S1. (**B, C**) EMSAs using 1 nM DNA probes with a monomeric FOXA1 element (**B**) or the composite DIV element (**C**) and a concentration series of the FOXA1 DBD. Lanes 1–14 are marked and contain decreasing FOXA1 DBD concentrations. 1: 2500 nM; 2: 1250 nM; 3: 625 nM; 4: 312.5 nM; 5: 156.3 nM; 6: 78.1 nM; 7: 39 nM; 8: 19.5 nM; 9: 9.8 nM; 10: 4.9 nM; 11: 2.4 nM; 12: 1.2 nM; 13: 0.6 nM and 14: no protein). DNA sequences are shown with core binding sites colored red. Dimer (D), monomer (M) and free DNA bands (F) are indicated. (**D**) EMSAs using DNA probes with DIV and CON motifs and mutants with inserted spacers (D2, C1 and C−1). 100 nM of each Cy5-labelled double stranded DNA was incubated with 400 or 320 nM of the FOXA1 DBD protein. (**E**) EMSAs using 100 nM DIV DNA (D0) and 1 to 10 base-pair spacers (D1 to D10) performed with 400 nM FOXA1 DBD protein. (**F**) Cooperativity values (ω) calculated after quantifying dimer, monomer and free DNA fractions from EMSAs (38,39). The mean ± SD of at least five measurements is shown (raw values in Supplementary Table S1).

FOXA1 binds as monomer to an AWTRTTKRYTY (where W: A/T, K: G/T, R: A/G and Y: C/T) consensus site (25) and several disease-associated single nucleotide polymorphisms (SNPs) were identified to map to this consensus and influence its binding affinity (26,27). This suggests that even a subtle perturbation to the cistrome of FOXA1 could contribute to disease progression. We have recently developed a new co-motif discovery algorithm termed TACO (Transcription factor Association from Complex Overrepresentation) that enables the discovery of cell-type specific TF dimer candidates from deep sequencing data even for overlapping position weight matrices (PWMs) (28,29). Using TACO, we found a strong enrichment of two palindromic FOXA1 motifs in DNase I hypersensitivity regions specific for breast and prostate cancer cells (29). In one motif the FOXA1 half-sites are arranged in a convergent orientation and were therefore termed ‘CON’ and in the second motif the half-sites are arranged in a compact diverging orientation and termed ‘DIV’ (Figure 1A). Subsequently, composite DNA elements resembling both CON as well as DIV motifs were recovered in ChIP-exonuclease sequencing data (30,31) as well as in high-throughput SELEX studies (32,33). These findings implied that FOXA1 and other *forkhead* factors often target genomic DNA as homodimers. TF homodimerization can profoundly influence the selection of target genes and increase binding affinity in particular by decreasing the dissociation rate (34). Moreover, changes in the quaternary structure of TF/DNA complexes can enable different outcomes regulated by one and the same TF in alternative genomic contexts. This has, for example, been demonstrated for Pit1-Oct-Unc (POU) TFs that recruit different co-factors depending on the DNA induced dimeric conformations (35–37). This way, a TF can act as repressor in the context of one dimeric conformation and as an activator in another. We therefore decided to study the novel FOXA1 dimer configuration using structural modeling, quantitative dimer assays, interrogation of genomic datasets and two types of reporter assays. We show that DIV DNA directs FOXA1 homodimerization in a highly cooperative fashion with strict constraints on half-site spacing. Structural models based on a crystal structure of FOXA3 bound to a non-canonical DNA element suggest that major structural adjustment would be required to enable direct interactions between juxtaposed FOXA1 molecules. FOXA1/DIV homodimer sites control chromatin binding, nucleosome dynamics and gene expression at critical FOXA1-dependent enhancers. Intriguingly, disease-associated SNPs disrupt or repress FOXA1 homodimerization and the comparison of dimer promoting versus dimer impeding alleles reveals differential regulatory activities.

## MATERIALS AND METHODS

### Purification of the FOXA1 DNA binding domain (DBD)

Protein production was performed using protocols established at the protein production platform (PPP) of the NTU, Singapore (https://www.proteins.sg/). Briefly, a pNic28-Bsa4 expression plasmid encoding the human FOXA1 DBD (spanning Asp158-Pro272 corresponding to 24.4% of the 472 amino acid full length protein; GenBank accession BC033890) was transformed into Rosetta (DE3) BL21 competent cells (Novagen) and grown in terrific broth (TB) medium at 37°C to an OD_600_ 0.6–0.8. Protein expression was induced with 0.2 mM isopropyl β-d-1-thiogalactopyranoside (IPTG) for 12 h at 25°C. After harvesting by centrifugation (8000 rpm for 15 min at 4°C), the bacteria were lysed by supersonication (400 W, 5 s for 99 times with 8 s gap between each time) in lysis buffer (100 mM HEPES ((4-(2-hydroxyethyl)-1-piperazineethanesulfonic acid), 500 mM NaCl, 10 mM imidazole, 10% glycerol, 0.5 mM TCEP (tris(2-carboxyethyl)phosphine) and 100 μM PMSF). The lysate was centrifuged again (10 000 rpm for 30 min at 4°C) to pellet the cellular debris while the supernatant was loaded onto a column containing Ni-NTA agarose beads (Thermo Fisher) equilibrated in lysis buffer. After washing with Buffer A (20 mM HEPES, 500 mM NaCl, 10 mM Imidazole, 10% glycerol and 0.5 mM TCEP, pH 7.5) and buffer B (20 mM HEPES, 500 mM NaCl, 25 mM imidazole, 10% glycerol and 0.5 mM TCEP, pH 7.5) for 5 times each, the target protein was eluted with elution buffer (20 mM HEPES, 500 mM NaCl, 500 mM imidazole, 10% glycerol and 0.5 mM TCEP, pH 7.5). Some batches were further purified by size exclusion chromatography using the AKTA express system and a Superdex 75 column (GE Healthcare). FOXA1 DBD alanine 232 mutations were introduced by PCR with Phusion polymerase (Thermo Fisher) using the circular pNic28-Bsa4 vector as template followed by DpnI digestion and verified by Sanger sequencing. Proteins were purified following the same procedures.

### Purification of full length FoxA1 protein

Full-length mouse FoxA1 protein (NM_008259.3) was cloned into a pET-28A vector containing a N-terminal His6 tag followed by a thrombin cleavage site (IGE, www.igebio.com). The protein expression was induced in Rosetta DE3 cells grown in Luria broth (LB) medium by adding 0.2 mM of IPTG. After 4 h, cells were harvested by centrifugation and resuspended in lysis buffer (5 mM imidazole, 20 mM Tris–HCl pH 7.9, 500 mM NaCl, 20 mM PMSF and 8 M urea) followed by 99 sonication cycles at 400 W for 5 s with 8 s breaks on ice. The lysate was centrifuged at 12 000 rpm for 30 min and the supernatant was incubated with 1 ml of Ni-NTA agarose beads (Thermo Fisher) and washed thoroughly. The protein was first eluted with elution buffer (400 mM Imidazole, 20 mM Tris, pH 7.9, 500 mM NaCl and 8M urea) and subjected to a step-wise refolding procedure by dialysis in the cold room: 6 M urea buffer (20 mM Tris pH 7.9, 500 mM NaCl and 6 M urea) for 1h, 4M urea buffer (20 mM Tris pH 7.9, 500 mM NaCl and 4 M urea) for 2 h, 2 M urea buffer (20 mM Tris pH 7.9, 500 mM NaCl and 2 M urea) for 2 h, urea-free buffer (20 mM Tris pH 7.9, 500 mM NaCl) for 2h and collected in storage buffer (20 mM Tris pH 7.9, 100 mM NaCl).

### EMSA (electrophoretic mobility shift assays)

5′ Cy5-labeled forward strand DNA oligo and their reverse complementary unlabeled strand (HPLC-purified, purchased from Invitrogen or BGI) were first annealed in 1× annealing buffer (20 mM Tris–HCl, pH 8.0; 50 mM MgCl_2_; 50 mM KCl) in a PCR block (Bio-rad) by heating to 95°C for 5 min and gradual cooling at 1°C/min to 16°C. 1-100 nM of dsDNA was incubated with varying concentrations of the FOXA1 DBD or of mouse FoxA1 full length (FoxA1 FL) protein in 1X binding buffer (20 mM Tris-HCl, pH 8.0; 0.1 mg/ml bovine serum albumin; 50 $\mu$M ZnCl_2_; 100 mM KCl; 10% (v/v) glycerol; 0.1% (v/v) Igepal CA630 and 2 mM β-mercaptoethanol) for 1 h at 4°C and 10 μl of sample was loaded onto pre-run 12% native polyacrylamide mini-gels (for FoxA1 FL 6% gels were used) in 1× Tris–glycine buffer (0.025 M Tris, 0.192 M glycine, pH 8.0) and electrophoresed for 30 min at 200 V in the cold room. The bands were detected with a Fuji FL-7000 scanner (GE Healthcare) using 635/670 nm excitation/emission wavelengths and band intensities were quantified using the ImageQuantTL software. Cooperativity factors were calculated as previously described (34,38,39). Dissociation constants (*K*_d_) from titration experiments with 1 nM DNA probe were estimated using non-linear curve fitting in R treating the DNA as fixed parameter as previously described (38). Sequences of all tested DNA elements are listed in Supplementary Table S1.

### Vector construction

For luciferase vector construction, 500 bp DNA fragments with central DIV motif candidates were cloned into a pGL4-TK luciferase reporter plasmid (Promega, E2241) using Phusion DNA polymerase (Thermo Fisher) or KOD-Fx-neo DNA polymerase (Toyobo). Restriction enzymes KpnI (NEB) and EcoRV (NEB) were used to digest the PCR products and the pGL4-TK backbone. Digested sequences were gel purified (Tiangen) and ligated using the T4 ligase (Takara). For constructing the Tol2 reporter vector (40), DIV motif candidates were digested with KpnI or BglII (NEB, forward sequence) and XhoI (NEB, reverse sequence) and then ligated. Full length human FOXA1 (gene ID: 3169) was PCR amplified using primers listed in Supplementary Table S1 and cloned into the pcDNA3 (41) vector using restriction enzymes EcoRI (NEB) and XhoI (NEB). Site-directed mutagenesis was carried out by amplifying the target plasmid with Phusion polymerase (Thermo Fisher) using overlapping primers encoding the mutated sequence followed by Dpn I (NEB) digestion. All DNA oligos are listed in Supplementary Table S1.

### Tol2 reporter assay (endogenous FOXA1 reporter assay)

MCF7 cells (ATCC) were grown in Dulbecco's Modified Eagle Medium (DMEM, Gibco) with 10% fetal bovine serum (FBS, Biowest) in 24-well plates to 50% confluency. Cells were transfected using 0.8 μl X-tremeGENE 9 DNA transfection reagent (Roche) containing 500 ng of Tol2 GFP reporter plasmid and 500 ng pCAGGS-TP Transposase expressing plasmid (40). After 72 h incubation, cells were trypsinized using 200 μl 0.25% Trypsin (Gibco), washed twice with 500 μl Dulbecco's Phosphate Buffered Saline (DPBS, Gibco) and resuspended in 500 μl DPBS. GFP fluorosecences was measured using Fluorescence-Activated Cell Sorting (FACS) method with a Calibur Flow Cytometer (BD Bioscience) using the GFP channel after gating for 10,000 living cells. Total GFP expression level of each tested sample was evaluated using the FlowJo software (FlowJo™). First, the median GFP signal of all 10 000 cells was recorded. Next, the percentage of GFP positive cells was determined after calibrating the gating using a Tol2 vector control without enhancer insert. The final expression value was calculated by multiplying median GFP expression with the fraction of GFP positive cells.

### Luciferase assay

Around 10^4^ HCT116 cells (ATCC) were plated in modified McCoy's 5A medium (Gibco) supplemented with 10% FBS (Biowest) in 24-well plates. Alternatively, 10^5^ T47D cells (ATCC) were plated in one well of 24-well plate in RPMI Media 1640 (Gibco). Both cell lines were grown for 24 h to reach 80% confluency. 200 ng luciferase reporter plasmids, 1 ng pRL-SV40 Renilla (E2231, Promega) and 50 ng pcDNA3-FOXA1 were mixed with 0.8 μl of X-tremeGENE 9 DNA Transfection Reagent (Roche) per well. After 24 h growth at 37°C and 5% CO_2_, cells were carefully washed with 500 μl (DPBS) and lysed with 100 μl 1× Passive Lysis Buffer (Promega). Dual Luciferase Reporter System (Promega) and a Veritas Microplate Luminometer (Turner Biosystems) were used to measure the Luciferase and Renilla activity following the manufacturer's instructions.

### Bioinformatics analysis

ChIP-seq data were downloaded from ENCODE (https://www.encodeproject.org) in narrowPeak format or from the Cistrome project (http://cistrome.org/Cistrome/Cistrome_Project.html). A subset of the datasets was re-processed using raw reads downloaded from the GEO followed by genome alignment with bowtie2 (42) and MACS for peak calling (43) with default parameters. Column 7 (signalValue) of the ENCODE narrow peak format was used as peak score in the boxplots. Accession numbers of data used in this study are listed in Supplementary Table S1. FOXA1 DIV (D) motif models are generated with composite TRANSFAC motifs M00791 and M01012 with the latter displaced with 4 bp offset using previously published data (29). Genome-wide motif coordinates were identified at a motif score threshold that provided 80% sensitivity in detecting ChIP-seq peaks. For the control motif coordinates (CON, C), spacers from +1 bp to +10 bp between two overlapped monomer motifs were added. Moreover, we excluded control loci that would overlap a ‘correctly spaced’ DIV dimer locus. As for the CON motif, we used the TRANSFAC motif M0102 twice, with the second motif reverse complemented and displaced by 9 bp. To obtain genome-wide coordinates for monomer locations we used FOXA1 monomer motif PWM provided by the HOMER database (AAAGTAAACA, discovered in a FOXA1 ChIP-seq study in MCF7 cells GEO accession GSE26831) and used the HOMER scanMotifGenomeWide.pl function with the hg38 genome.

Intersections between ChIP-seq peaks and motif coordinates were done using bedtools (http://bedtools.readthedocs.io/en/latest/, (44)) functions intersectBed or windowBed (window 100 bp) or foverlaps of the data.table package (https://cran.r-project.org/web/packages/data.table/). ATAC-seq data were downloaded (GEO accession number GSE84515) and converted into fastq using fastq-dump. Bowtie2 was used to align the reads to the hg38 genome build followed by further processing and file format conversions using samtools. These files were used for coverage analysis over ChIPseq peak categories defined by matches to FOXA1 motif types. EAseq v.1.04 (45) was used to draw ATAC-seq and ChIP-seq read coverage heat maps normalized by reads-per-million. R packages data.table and ggplot2 where used for analysis and visualization using custom scripts.

### Modeling of FOXA1 dimer structures

Models of FOXA1 homodimers on DIV DNA with 0 to 10 bp spacer were prepared by using a crystal structure of a HNF-3γ (FOXA3) *forkhead*/DNA complex (protein data bank (PDB) accession: 1VTN) (22). The amino acid sequence of the FOXA3 portion present in the crystal structure is 96% identical with the human FOXA1 DNA binding domain (UNIPROT ID: P55317). The DNA sequence bound to FOXA3 in the crystal structure is 5′-G_1_G_2_T_3_T_4_G_5_-3′/3′-C’_1_C’_2_A’_3_A’_4_C’_5_-5′, which deviates from the FOXA1 consensus 5′-T_1_A_2_T_3_T_4_T_5_-3′/3′-A’_1_T’_2_A’_3_A’_4_A’_5_-5′. We used the conserved binding of Asn165 with A’_4_ via a bi-dentate H-bond as quality control criterion to validate our dimer models and also to construct alternative models with switched contacts. The strategy to model FOXA1 dimers on differently spaced elements or with switched Asn165-Adenine contacts on the DIV (D0) element is graphically explained in Supplementary Figure S1A.

First, binary FOXA1/DNA complexes were prepared by comparative homology modeling (https://www.salilab.org/modeller) using the template of the FOXA3 crystal structure. The DNA element was considered as rigid body during homology modeling. Next, we designed DNA elements with canonical B-DNA geometry containing half sites matching the sequences in 1VTN to facilitate superposition of the binary FOXA1/DNA on forward and reverse strands of the ideal B-DNA. The sequence CAAC was used for superposition to obtain the initial FOXA1 dimer models and adjacent bases were concatenated. Next, nucleotides of the DIV from the *PVT1* enhancers were used to replace nucleotides of the initial models to construct a model with a consensus DIV sequence (Supplementary Figure S1B and C). The energy of the ternary complex models was minimized by using amber force fields ff14SB for the proteins (46) and amber force fields FF99BSC0 (47) for DNA with the bidentate Asn165—adenine hydrogen bond defined as positional restraint. Superposition models were prepared by VMD and figures were generated using both Chimera 1.11 (48) (https://www.cgl.ucsf.edu/chimera/) and VMD1.9.3 (https://www-s.ks.uiuc.edu/Research/vmd/vmd-1.9.3/).

## RESULTS

### FOXA1 forms a highly cooperative homodimer on a compact DNA element

By scoring for the enrichment of co-occurring position weight matrices in cell-type-specific DNase hypersensitive (HS) regions in 78 human cell lines, we previously identified candidate TF dimer configurations that could contribute to their cell-type specific functions (28,29). Two palindromic FOXA1 motifs constituted top hits of this analysis. First, a compact composite motif termed ‘diverging’ DIV (D0) motif was found where the AT-rich core of the *forkhead* motif (TATTT) overlap so that the central TA dinucleotide is shared by juxtaposed half-sites (AAATATTT). We also identified a less compact composite motif with *forkhead* motifs arranged in alternative directions which we called ‘converging’ or CON (C0) motif (Figure 1A). We were interested in both motifs for their strong enrichment in the breast cancer cell line MCF7 and prostate cancer cell line LNCaP.

To test whether FOXA1 homodimerises on these sequences, we established quantitative electrophoretic mobility shift assays (EMSAs) using the purified DNA binding domain (DBD) of FOXA1 (henceforth termed FOXA1). In the absence of DNA, FOXA1 does not dimerize but forms a monodisperse monomer as judged from calibrated size-exclusion chromatograms (Supplementary Figure S1D). We first performed EMSAs with 1 nM DNA probe encoding the FOXA1 monomer element and a concentration series of FOXA and measured a mean *K*_d_monomer_ of 2.4 ± 1.06 nM (*n* = 3, Figure 1B). Similar titrations in the presence of DIV DNA showed that dimeric FOXA1 bands begin to form at the lowest FOXA1 concentrations (0.61 nM) indicating a dimerization with strongly positive cooperativity (Figure 1C). An apparent binding affinity *K*_d_app_ for the binding of FOXA1 to DIV DNA was determined to be 4.5 ± 1.6 nM by regarding monomeric and dimeric states as an overall bound DNA fraction. To quantify the efficiency of the FOXA1 to homodimerize, we next estimated the cooperativity factor (ω) under equilibrium conditions at a DNA concentration of 100 nM. These measurements provide ratios of equilibrium binding constants (ω = *K*_d_monomer_/*K*_d_dimer_). Here, *K*_d_dimer_ represents the dissociation constant for the binding of a second FOXA1 molecule to a pre-formed FOXA1/DNA complex. Thus, cooperativity factors indicate how two TF molecules influence their mutual occupancies on a given DNA element with composite binding sites (34,38). If ω > 1, TFs bind with positive cooperativity; if ω<1, TFs bind with negative cooperativity or, in other words, compete and if ω = 1 binding is independent or non-cooperative (49). Using these assays, we compared the binding of FOXA1 to co-motifs enriched in DNase-HS regions (D0/DIV and C0/CON motifs) with control elements where the spacing between half-sites was altered (D2, C1 and C-1). FOXA1 binds DIV DNA with highly positive cooperativity (ω = 56.3 ± 11.9; Figure 1D–F, Supplementary Figure S1E, Supplementary Table S1). For the C0 DNA a profoundly lower cooperativity factor was measured (ω = 1.8 ± 1.3). If the half-site spacing is perturbed, the dimerization is impeded for both configurations of DNA elements. To further dissect the reliance of cooperative binding on half-site spacing, we inserted spacers from 1 to 10 bp between the half sites of the DIV (Figure 1E and F, Supplementary Figure S1F, Supplementary Table S1). This experiment revealed that cooperative dimerization of FOXA1 on DNA strictly depends on compact half-site spacing. Addition of spacers separating the FOXA1 core motifs decreased ω by at least an order of magnitude and in the case of the D3 configuration completely obliterated the formation of dimeric complexes. While the *forkhead* DBD is sufficient for cooperative dimerization, this binding mode is also retained in the context of recombinantly purified full-length mouse FoxA1 protein (Supplementary Figure S1G).

### Structural modeling suggests alternative mechanisms for the dimer formation

To understand the basis for FOXA1 dimerization on DIV DNA, we constructed a series of FOXA1 DBD dimer models. First, we used a published FOXA3 DBD-DNA crystal structure (PDB ID 1VTN (22)) to produce FOXA1 homology models. The FOXA3 DBD spans about 25% of the full-length protein and differs by five amino acids from the FOXA1 DBD. Next, dimer models were generated by the superposition of two binary FOXA1/DNA models onto ideal B-DNA templates containing DIV sequences (Supplementary Figure S1A-C). To maintain the experimental DNA curvature, we concatenated experimental DNA fragments followed by removal of the B-DNA template. Finally, the complex models were energy minimized allowing for structural adjustments of both the protein and the DNA components leading to models with curved DNA. Notably, the DNA element of the 1VTN (CGTTG) model differs at several key positions from the *forkhead* consensus (TATTT) (Supplementary Figure S1B). Therefore, the curvature of the DNA of a binary complex with FOXA1 may not be correctly represented in presently available models. Further, profound structural changes of the DNA could accompany the assembly of dimeric complexes.

**Figure 2.**
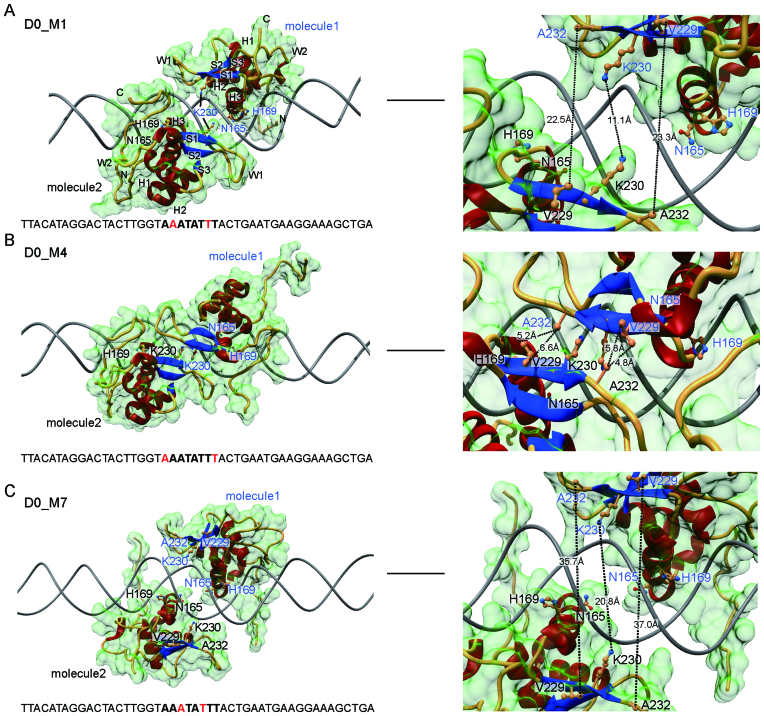
Structural model of dimeric FOXA1/DNA complexes. Structural models were constructed using FOXA1 homology models generated using the FOXA3/DNA crystal structures (PDB ID 1VTN) as template. The modeling strategy is outlined in Supplementary Figure S1A–C. The interaction of Asn165 with an Adenine is a critical mediator of the DNA recognition of *forkhead* DBDs. Based on the alignment with binary *forkhead* DBD/DNA structures Asn165 is expected to interact with A_4_’ in both 5′-T_1_A_2_T_3_T_4_T_5_-3′/3′-A’_1_T’_2_A’_3_A’_4_A’_5_-5′ DIV half sites (**A**, D0_M1). We surmised that in the context of a homodimeric complex Asn165 could switch Adenines leading to alternative models where Asn165 contacts A_5_’ (**B**, model D0_M4) or A_3_’ (C, model D0_M7). Left panels show overviews and right panels are zoomed in views highlighting amino acids V229 and A232 exposed to the neighboring molecule that could mediate the dimer formation. The DNA is shown as gray tube, protein helices, sheets or loops are in red, blue and yellow cartoons, respectively. The molecular surface is shown in transparent green. Selected amino acids are labeled and shown as ball-and-sticks. The DNA sequence used to construct the models is shown and nucleotides (or their reverse-complement) contacted by Asn165 are in red.

A conserved feature in *forkhead* DBD/DNA complexes is a bidentate hydrogen bound of Asn165 with Adenine A’4 (5′-T_1_A_2_T_3_T_4_T_5_-3′/3′-A’_1_T’_2_A’_3_A’_4_A’_5_-5′) (Supplementary Figure S1A). We first performed superpositions that maintain the interaction of Asn165 with A’4 in the final complex models. In these models we could not observe any intermolecular protein-protein interactions between juxtaposed FOXA1 molecules suggesting that the dimer formation could be facilitated allosterically through DNA (Figure 2A). However, as the DIV is AT-rich we surmised that in the context of a dimeric complex Asn165 could be induced to switch Adenines and bind to A’_3_ or A’_5_ instead. To test this possibility, we constructed nine alternative models with all possible combinations of Asn165 with Adenines 3–5 in either half site (Supplementary Figure S1A, B). Out of the nine models three are symmetric, that is, Asn165 binds the same Adenine in either of the two half-sites (Figure 2A–C). Out of the three symmetric models for FOXA1 homodimer, the D0-M4 model (where Asn165 binds A’_5_) shows and extensive hydrophobic protein-protein contact interface formed predominantly by Val229 and Ala232 (Figure 2B and Supplementary Figure S1C). We also generated structure models for all DIV control motif configurations with spacer lengths 1–10 (Supplementary Figure S2A, B). In this set of models, severe clashes were observed for the D3 configuration (Supplementary Figure S2B) consistent with the absence of any dimer band in EMSAs (Figure 1E). Remaining configurations showed moderate structural clashes, which could possibly be relieved by conformational adjustments of the protein and changed shape of the DNA (Supplementary Figure S2A).

The D0-M4 model (Figure 2B) appears similar to the non-cooperative D2 model that was generated by separating the otherwise overlapping FOXA1 half-sites of the DIV (Supplementary Figure S2A). However, a superposition between the 2 models reveals that the beta-sheets of the FOXA1 monomers are closer to each other in the D2 model, potentially leading to a suboptimal interaction interface. Moreover, the DNA sequence differs between the two models, leading to alternative DNA shapes that may cause modifications of the predicted protein–protein interaction interface (Supplementary Figure S2A). Thus, the D0_M4 model cannot be invalidated due to the lack of positive cooperativity on the D2 element (Figure 1D–F). We propose that D0_M1, D0_M4 and D0_M7 all represent potentially valid initial models for the FOXA1 dimerization on the DIV motif based on the currently available data. Notably, only the D0_M1 model has the direct readout of the DNA sequence similar to that observed for the consensus sequence. Further validation of the models with experiments and molecular dynamics simulation is needed.

Notably, an Ala232Val mutation has been reported to drive prostate cancer (50). We decided to test whether mutations to residue Ala232 influence cooperative dimerization on the DNA. However, mutations associated with prostate cancer and 10 other substitutions only mildly influence the cooperativity on the DIV sequence (<1.5-fold change to ω, Supplementary Figure S2C). As a more rigorous test we also constructed double mutants where both of the putative interface residues Val229 and Ala232 were concurrently mutated to acidic glutamates (Val229Glu/Ala232Glu and Val229Arg/Ala232Arg). EMSAs showed that the Val229Arg/Ala232Arg double mutation does not influence DNA binding (Supplementary Figure S2D). However, surprisingly, the Val229Glu/Ala232Glu double mutations lead to a marked increase in the cooperativity although the affinity for monomeric binding is reduced.

In the absence of experimental structures of a ternary FOXA1/DIV complex, the curvature of the bound DNA, the protein-DNA contact interface and the structural basis for dimerization remains hypothetical. The mechanism for the cooperative dimer formation could be due to two basic mechanisms. First, direct protein-protein interactions that would require major structural adjustments and including contact interface switching or the deformation of DNA. Second, DNA-mediated allosteric mechanism could facilitate cooperative DNA recognition by FOXA1. Such mechanisms have been described for a growing number of TF dimer pairs and appear to be common theme in TF biology (51–53).

### FOXA1 strongly binds to DIV loci in human cancer cells

**Figure 3.**
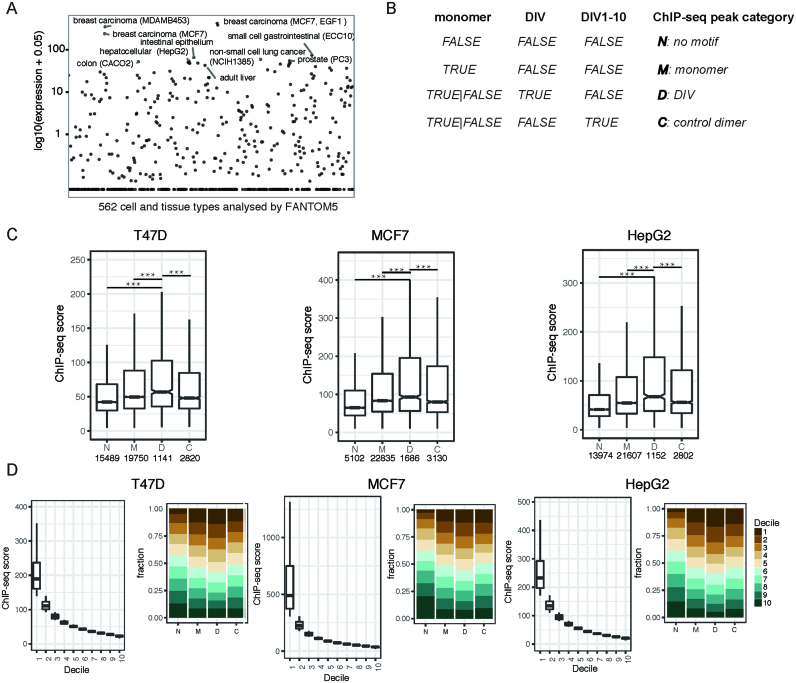
FOXA1 strongly binds to DIV sequences in the context of chromatin. (**A**) FOXA1 expression measured by the FANTOM 5 consortium in 562 cell and tissue types. Each dot represents a cell or tissue type and selected samples with highest FOXA1 expression are marked. (**B**) Schematic how ChIP-seq peaks were categorized based on the absence/presence of monomer, DIV or control dimer (DIV1-10) motifs. (**C**) Boxplot to compare ChIP-seq scores (ENCODE narrowPeak signal values) in the four FOXA1 ChIP-seq peak categories defined in (B) using data from T47D, HepG2 and MCF7 cells. *P*-values are calculated using pairwise comparisons with the unpaired Wilcoxon rank sum test (R function pairwise.wilcox.test) and adjusted using the Holm method (***P < 0.001). (**D**) ChIP-seq peaks were ranked by signal values and divided into deciles (top decile = 1, bottom decile = 10, shown as boxplots) and the fractional counts of the four binding categories per decile are shown as proportional barplots.

FOXA1 is a master regulator of endodermal cell and tissue types but also associated with tumorigenesis in several cancers including breast, prostate and liver cancer ((9), reviewed in (8)). Accordingly, FOXA1 expression is highest in normal liver and intestine epithelium as well as breast, prostate, gastrointestinal and liver cancer cell lines amongst a panel of 562 samples analyzed by the FANTOM5 consortium (54) (Figure 3A). To study the relevance of the DIV to define the genomic binding profiles of FOXA1, we next defined four categories of binding locations in publicly available ChIP-seq datasets (Figure 3B). Category ‘D’ contains sites with matches to the DIV PWMs in the peak region (1.1e+5 instances in the human genome hg38, ‘D’). Category ‘C’ are control dimer sites that have matches to motifs where the dimer promoting configuration is disrupted by introduction of 1–10 spacers (D1-D10, 5.1e+5 loci in hg38, Supplementary Figure S3A). Category ‘C’ controls for the preference for a specific configuration but maintains the number of half sites similar to DIV locations. Next, we defined locations where FOXA1 binds exclusively as monomer (‘M’). Lastly, remaining locations are designated ‘N’ for ‘no motif’ for the lack obvious matches to any of the three types of FOXA1 motifs (Figure 3B). FOXA1 ChIP-seq datasets from MCF7, T47D and HepG2 cells typically contain 1000–2000 matches to the DIV and show an enrichment for the DIV as compared to the dimer control with respect to the genome-wide DIV/control ratio (Supplementary Figure S3B). As a proxy for cooperative dimerization in a cellular context, we compared the ChIP-seq signals over the four classes of binding sites. FOXA1 loci with DIV signatures in T47D, MCF7 and HepG2 cells exhibit significantly stronger ChIP-seq read intensities compared to regions with alternative motif matches (Figure 3C, Supplementary S3C). As an alternative analysis we ranked FOXA1 ChIP-seq peaks in MCF7, T47D and HepG2 cells by signal strength and divided them into deciles (Figure 3D). Next we quantified the fractional occurrence of the four binding categories per decile (Figure 3D). This analysis shows that FOXA1/DIV peaks are concentrated in the top deciles to a larger degree than the three other binding categories.

We also inspected ChIP-seq signals for alternative chromatin associated factors GATA3, the co-activator Histone acetyltransferase P300 (EP300), CTCF and c-Jun (Supplementary Figure S3E). We did not observe increased signals over DIV sequences as compared to other binding site categories indicating that the effect is specific for FOXA1. We conclude that FOXA1 associates with the DIV more strongly than with monomeric or alternative dimer sites in a chromatin context because of the highly cooperative homodimerization promoted by this binding site. We next performed gene ontology analysis using gene sets linked to FOXA1/DIV binding events in T47D or MCF7 cells with signatures of changed expression after chemical or genetic perturbation (Supplementary Figure S3E). Amongst the top most significantly enriched gene sets bound by FOXA1/DIV are genes up-regulated in xenografts resistant to endocrine therapy (55), differentially upregulated in luminal as compared to basal or mesenchymal breast cancer cell lines (56), down regulated in breast cancer cells depleted of ESR1 (57) and other gene sets associated with response to nuclear receptor signaling and oncogenic pathways. This suggests that genes associated with FOXA1/DIV locations are sensitive to perturbation of pathways relevant to cancer progression in particular in breast cancer models.

### FOXA1 homodimerizes on DIV motifs to regulate enhancers near genes implicated in cancer progression

**Figure 4.**
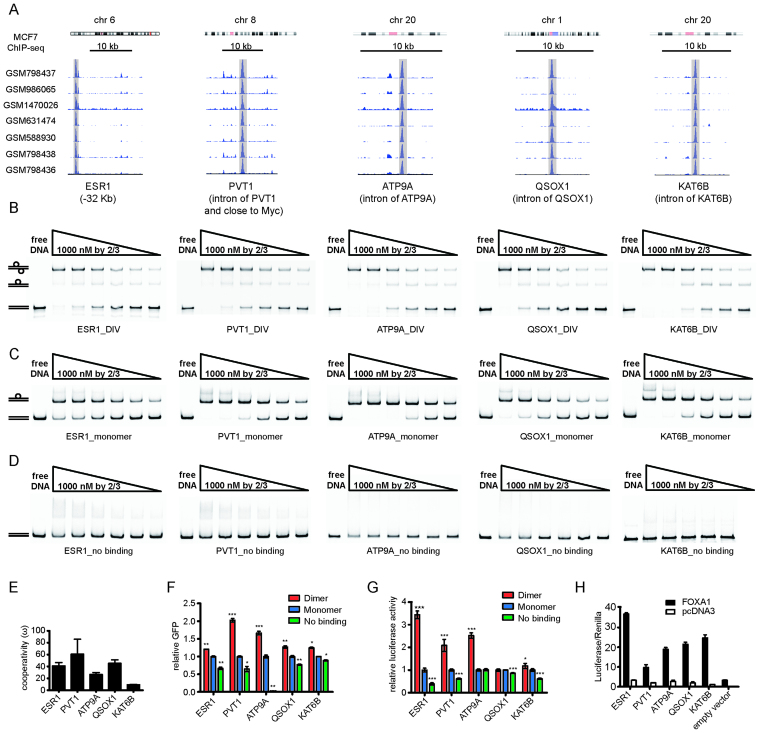
FOXA1/DIV binding regulates gene expression in cancer cells. (**A**) FOXA1 ChIP-seq peaks in MCF7 cells (accession numbers indicated) at five DIV loci near genes with potential roles in oncogenesis (see also Supplementary Figure S4A and B). (**B**) EMSAs using Cy5 labeled DNA elements derived from the five endogenous DIV loci. (**C**) EMSAs where the five DIV DNA elements were mutated to monomer binding sites by adding a 3 base-pair spacer destroying dimeric binding but leaving monomeric binding intact (‘Monomer’). (**D**) EMSA where both half sites of the DIV elements were mutated abolishing binding (‘No binding’). (**E**) Homodimer cooperativity value ($\omega$) shown as mean ± SD from *n*$\geq$ 3 measurements. (**F**) Total GFP fluorescence quantified by FACS analysis using Tol2 constructs containing DIV enhancers or the two types of mutated DIV sites (‘Monomer’ or ‘No Binding’) integrated into the genomes of MCF7 cells. FACS plots and photographs of cells are in Supplementary Figure S4D. (**G**) Dual Luciferase reporter assay using the T47D cell line endogenously expressing FOXA1. (**H**) Dual Luciferase assay in HCT116 cells co-transfected with FOXA1 expression plasmids (filled bars) or the pcDNA3 control (empty bars). Empty vector is the luciferase reporter without inserted DIV enhancer. Reporter signals in (F) and (G) were normalized to ‘Monomer’ values. Luciferase and Tol2 reporters were constructed as outlined in Supplementary Figure S4C. The mean ± SD of three biological replicates is shown in (F), (G) and (H). *P*-values were calculated using the unpaired two-tailed Student's *t*-test (***P< 0.001; **P< 0.01;*P< 0.05).

We next selected five FOXA1/DIV loci associated with genes responsive to perturbation studies that reproducibly showed strong ChIP-seq signals in MCF7 cells (Figure 4A, Supplementary Figure S3E). These loci are located either in introns or within 50 kb upstream of the TSS of genes with a strong expression in MCF7 cells and other relevant cell and tissue types (Supplementary Figure S4A and B). Moreover, these genes were reported to play critical roles in cancer or essential cellular processes including *ESR1* (58), *PVT1/MYC* (59), *ATP9A* (60), *QSOX1* (61) and *KAT6B* (62) (Supplementary Figure S4A). *PVT1* encodes for a long non-coding RNA that resides in the 8q24 locus shared with the oncogene *MYC* (63). This locus is strongly amplified across a panel of malignant cancers and a marker for poor prognosis (64,65). We performed EMSAs using sequence derived from these five endogenous loci as well as two types of mutants (Figure 4B–D). First, we engineered these loci by introducing 3 bp spacers between the half sites reminiscent to the D3 element that is incompatible with dimeric binding (Figure 1E, ‘Monomer’, Supplementary Table S1). Moreover, we mutated both half-sites to generate DNA elements with completely destroyed FOXA1 consensus (‘No binding’, Supplementary Table S1). Results show a strongly positive cooperativity for FOXA1 homodimerization on all five DIV sequences with the highest ω value for the *PVT1/MYC* locus (Figure 4B–E). Introduction of the 3bp spacer destroys dimeric binding but leaves the affinity for monomeric binding unaffected (Figure 4C). Degeneration of both FOXA1 half-sites abolishes FOXA1 binding almost completely at the concentrations tested (Figure 4D). To test whether binding with purified components is associated with gene regulation in a context of cells and chromatin, we designed two reporter assays to validate the enhancer activity of these loci. First, we cloned ∼500 bp fragments encompassing the FOXA1-bound DIV sequences tested by EMSA into Tol2 vectors. The Tol2 system enables transposase-mediated integration into the genome of MCF7 cells that strongly express FOXA1 (Supplementary Figure S4C). Additionally, we designed a luciferase reporter assay. The latter was performed in T47D cells expressing FOXA1 endogenously and in the colon cancer cell line HCT116 where FOXA1 is not normally expressed (66) (Supplementary Figure S4C). The expression levels of the Tol2-GFP reporters driven by the various enhancer constructs were quantified in MCF7 cells using FACS and expression was scored taking the fraction of GFP positive cells as well as the median GFP signal into account (Supplementary Figure S4D). These expression levels show that sequences containing DIV motifs exhibit a strong enhancer activity (Figure 4F, ‘Dimer’). The expression of GFP reporters where the DIV was mutated so FOXA1 can only bind monomerically (‘Monomer’) or with two disrupted half-sites (‘No binding’) showed a significantly depleted reporter activity suggesting cooperative dimerization is required for full reporter activation (Figure 4F). Analogously, luciferase reporter activity was reduced in T47D cells for 4 out of 5 sequences when the homoderimic binding was disrupted (Figure 4G). Lastly, luciferase reporter activity was strongly elevated in HCT116 devoid of endogenous FOXA1 upon the exogenous provision of FOXA1 indicating that reporter activation is FOXA1-dependent (Figure 4H). Collectively, these results show that FOXA1 dimerizes cooperatively on endogenous DNA sequences and that the dimeric FOXA1/DIV configuration is required for the efficient activation of these enhancer sequences.

### DIV sequences mediate chromatin opening at locations pre-bound by FOXA1 upon inhibition of the PI3K pathway

As FOXA1 is designated as a hallmark pioneer TF, we next asked whether the FOXA1/DIV configuration is involved in the regulation of chromatin accessibility. To address this question we became interested in a study that explored chromatin changes in the breast cancer cell line T47D in response to treatment with the phosphatidylinositol 3-kinase (PI3K) inhibitor BYL719 (henceforth termed BYL) (67). The PI3K pathway is hyperactive in ∼70% of breast tumors and therefore an attractive target for anti-cancer therapies (68). However, PI3K kinase inhibition can lead to a potent compensatory response and cancer relapse, presumably facilitated by ERα-associated regulatory programs. To study the molecular mechanism for this process, Toska and colleagues compared the binding profile of FOXA1 and the chromatin accessibility measured by ATAC-seq in the absence and presence of BYL (67). Intriguingly, the authors reported a motif to become enriched in the FOXA1 binding landscape after BYL719 treatment representing a perfect match to the DIV. However, the authors refer to this motif as ‘Homeobox’ motif because of missing annotations of the DIV in common motif databases. We therefore decided to probe whether FOXA1/DIV configurations contribute to the compensatory chromatin remodeling in response to PI3K pathway inhibition.

**Figure 5.**
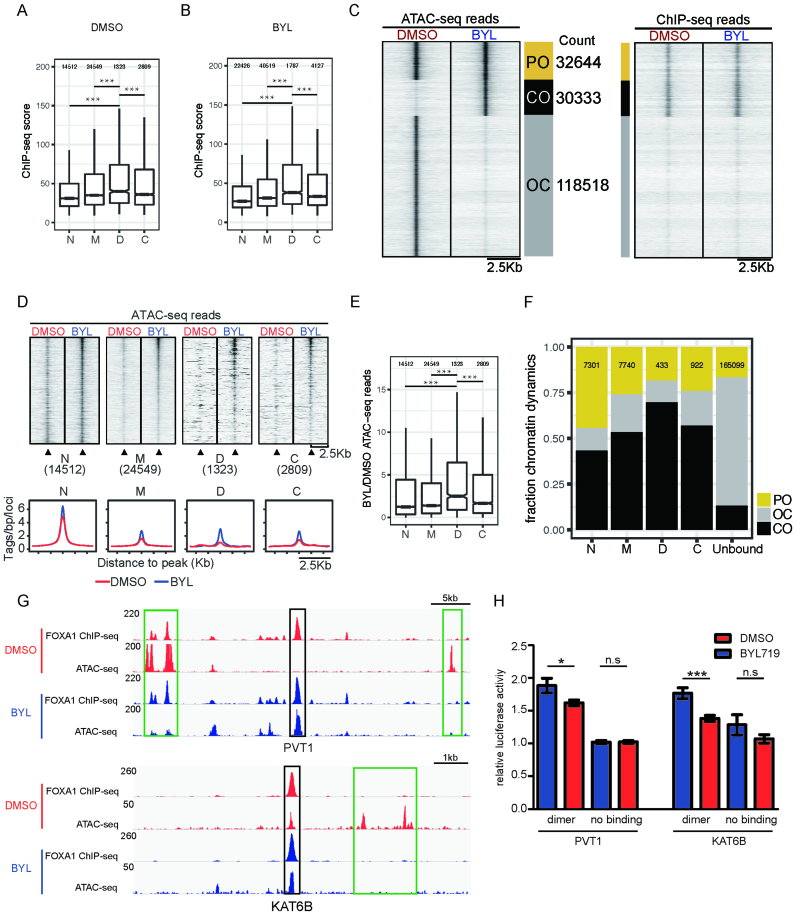
FOXA1 bound DIV locations are associated with chromatin dynamics. (**A, B**) Boxplot of ChIP-seq scores (summit height of fragment pileup) from FOXA1 data in T47D cells comparing peak categories for the subsets containing DIV motifs (D), control motifs (C), monomer motifs (M) and no FOXA1 motif (N) (see Figure 2B). T47D cells were exposed to DMSO (A) or to the PI3K pathway inhibitor BYL719 (B) (67). (**C**). Heatmap of ATAC-seq reads as well as FOXA1 ChIP-seq reads under DMSO or BYL719 treatment conditions in three categories of accessibility patterns: PO (permanently open in DMSO and BYL), CO (closed in DMSO but open in BYL) and OC (open in DMSO but closed in BYL). (**D**) ATAC-seq read heatmaps under DMSO or BYL719 treatment conditions facetted by the four FOXA1 ChIP-seq peak categories defined in the DMSO condition. The lower panels are aggregate pileups of ATAC-seq signals. (**E**) Boxplot of the ratio between ATAC-seq read counts between BYL719 treated T47D cells and non-treated (DMSO) T47D cells in the four categories of FOXA1 binding sites. (**F**) Fractional barplots showing the relative proportions of FOXA1 peak categories and sites not bound by FOXA1 (‘Unbound’) associated with PO, OC or CO sites defined according to accessibility patterns measured by ATAC-seq (see panel C). (**G**) Genome browser plot examples of ChIP-seq and ATAC-seq signals at FOXA1/DIV sites near the *PVT1/MYC* and *KAT6B* loci. Black boxes mark locations with DIV motif while green box show alternative location showing a disappearance of ATAC-seq signals. (**H**) Bar plot of the Luciferase/Renilla signal measured in T47D cells treated with DMSO (red) or BYL719 (blue) using DIV element containing luciferase reporter from the *PVT1/MYC* and *KAT6B* loci and mutated ‘no binding’ controls. The mean ± SD of 3 biological replicates is shown. *P*-values in (H) were calculated using the unpaired two-tailed Student's *t*-test (***P< 0.001; **P< 0.01; *P< 0.05). *P*-values in (A), (B) and (E) are calculated using Wilcoxon rank sum test and adjusted using the Holm method ( ***P< 0.001).

Peaks of the DIV category (‘D’) exhibit stronger ChIP-seq signals than loci with monomeric sites (‘M’), sites with perturbed dimer configurations (‘C’) and sites without detectable FOXA1 consensus element (‘N’) under both DMSO and BYL conditions (Figure 5A and B). We grouped genomic locations according to the closed-open dynamics measured by ATAC-seq in the absence or presence of PI3K inhibition. Permanently open (PO) sites are open under both DMSO and BYL conditions; close-to-open (CO) sites gain accessibility and open-to-close (OC) sites loose accessibility in response to BYL treatment (Figure 5C). We found that majority of loci belong to the OC category indicating lost accessibility upon BYL treatment. However, the FOXA1 ChIP-seq signal is predominantly associated with the PO or CO categories but barely detectable in the OC category (Figure 5C). This suggests that the absence of FOXA1 sensitizes genomic locations for closing whilst the presence of FOXA1 could have two roles. First, FOXA1 could function to maintain the open chromatin state of PO sites. Second, closed sites pre-bound by FOXA1 could become open in response to PI3K inhibition. We next inspected ATAC-seq signals over locations pre-bound by FOXA1 under DMSO conditions. We found that locations without matches to *forkhead* binding motifs (‘N’) are mostly pre-opened under DMSO conditions and show only a marginal increase in ATAC-seq signals after BYL treatment (Figure 5D and E, Supplementary Figure S5A). However, strikingly, FOXA1/DIV locations pre-bound at DMSO conditions are mostly closed but show a strong increase in ATAC-seq signal following PI3K pathway inhibition (Figure 5D and E, Supplementary Figure S5A). Consistently, a high proportion of FOXA1/DIV sites bound under DMSO conditions maps to locations of the CO category (Figure 5F). Whilst, FOXA1/C and FOXA1/M sites also show a preference for CO locations this association is more significant for FOXA1/DIV sites (Fisher's exact test *P* = 2.4e–05 (DIV versus control dimer)). We conclude that locations pre-bound by FOXA1 under DMSO conditions are subject to a closed-to-open transition upon PI3K inhibition and this effect is most profound for the subset of FOXA1/DIV location. The *PVT1/MYC* and *KAT6B* loci illustrate this effect with equally strong ChIP-seq signals under DMSO and BYL719 conditions but a strong increase of ATAC-seq signal after PI3K inhibition (Figure 5G). We next tested whether the DIV loci near the *PVT1/MYC* and K*AT6B* genes respond to PI3K inhibition in our luciferase reporter assay. Sequences with intact DIV elements show an elevated reporter activity in response to BYL treatment whilst sites with mutated DIV elements show no significant response (Figure 5H). However, plasmid-based reporter constructs are unlikely to exactly resemble the chromatin configurations of the endogenous loci. Therefore, we cannot be certain that the observed changes to reporter activity are due to the same chromatin closed-open dynamics apparent in the ATAC-seq data. Nevertheless, this analysis suggests that of blocking the PI3K pathway leads to chromatin remodeling at pre-bound FOXA1/DIV locations that could impact the transcriptional output.

### Disease-associated SNPs within the DIV motif induce allele-specific dimer formation and expression activity

**Figure 6.**
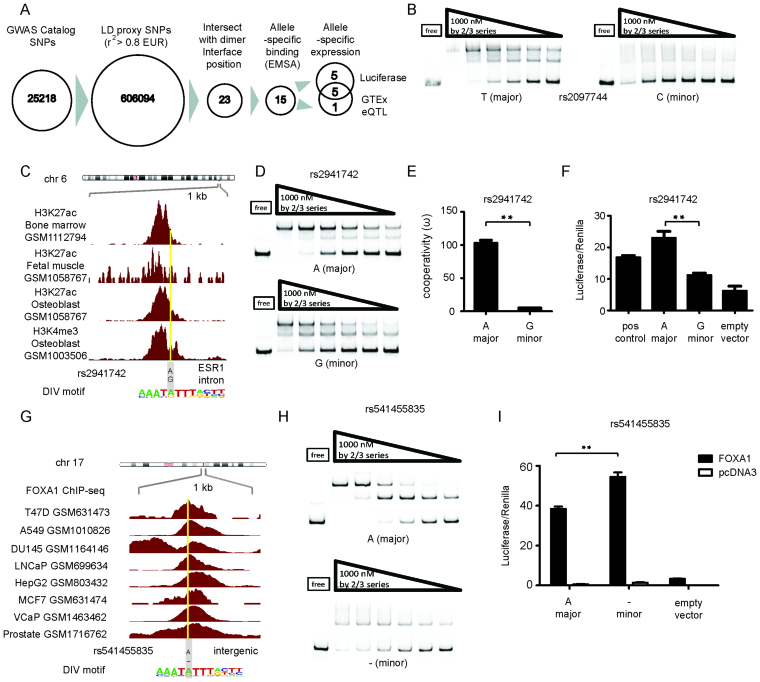
Disease-associated SNPs perturb dimerization and gene expression. (**A**) Flowchart to select SNPs for functional evaluation. (**B**) EMSAs for the rs2097744 locus where the minor allele completely disrupts dimerization. (**C**) ChIP-seq profiles of histone marks at the rs2941742 locus in various cell lines. (**D**) EMSA comparing dimer formation for major and minor alleles of rs2941742. (**E**) Cooperativity factor for EMSA in D (mean ± SD, *n* = 5). (**F**) Dual luciferase assay with exogenously supplied FOXA1 in HCT116 cells using both alleles of rs2941742 (mean ± SD, *n* = 5). (**G**) FOXA1 ChIP-seq profiles from several human cancer cell lines at the rs5414555835 locus (an alternative ID of the same locus is rs67668514). (**H**) EMSAs using the major and the minor allele of rs5414555835. I. Dual luciferase assay for the two alleles of the rs5414555835 locus in HCT116 cells. Filled bars are for exogenously provided full length FOXA1 and empty bars for the pcDNA3 vector controls (mean ± SD, *n* = 3 biological replicates). *P*-values were calculated using the unpaired two-tailed Student's *t*-test (**P< 0.01).

We surmised that cooperative FOXA1 homodimerisation on DIV sequences is necessary to execute a functional outcome on a subset of genomic loci. If the dimer motif is perturbed, a locus could loose the ability to effectively recruit FOXA1. Or, alternatively, monomeric FOXA1 could be unable to trigger a regulatory response despite effective recruitment. As a consequence, aberrant cellular responses could be evoked contributing to human diseases. To test this hypothesis we interrogated genome-wide association studies (GWAS) for variants affecting FOXA1 dimerization. We considered 25 218 disease-associated SNPs available from public resources (http://www.gwascentral.org, data released January 2016) and extracted further SNPs in linkage disequilibrium with them (proxy LD SNPs) using Haploreg (http://archive.broadinstitute.org/mammals/haploreg/haploreg.php) with parameter *r*^2^ > 0.8 in the European population. This way, we obtained a set of 606 094 candidate SNPs. We then intersected SNP coordinates with genome-wide DIV coordinates affecting the central TA site (AAATATTT), which we assumed to be most critical for the cooperative homodimerization of FOXA1. This way, we obtained 23 candidate SNPs whose minor allele may perturb FOXA1 homodimerization and *forkhead*-dependent transcriptional networks. To determine whether FOXA1 exhibits allelic differences in dimer formation, we performed EMSAs on all 23 SNP candidates with either major or minor allele sequences. Using this strategy, we identified 15 SNPs that profoundly perturb FOXA1 dimerization (Figure 6A, Table 1, Supplementary Figure S6A). We further inspected each of the 15 SNPs in the dataset provided by the genotype tissue expression (GTEx) consortium (https://gtexportal.org/) and found that six of the SNPs or their LD SNPs constitute GTEx expression quantitative trait locus (eQTLs, Figure 6A, Table 1). In some instances, e.g. rs2097744 that is associated with non-small cell lung cancer, the minor allele completely disrupted dimeric FOXA1 binding (Figure 6B). In other cases, the minor alleles decreased the cooperativity of FOXA1 dimerization (Supplementary Table S1). We next focused on SNPs supported by genomic annotations in tissues relying on the activity of FOXA1 or related *forkhead* family proteins.

**Table 1.** 

SNP ID	Location (hg38)	Gene	Disease association	Annotation	Luciferase		GTEx
					Major	Minor	eQTL
rs2858870	chr6:32604474	HLA-DRB1	Nodular sclerosis Hodgkin lymphoma	Promoter histone marks, enhancer histone marks, and DNase marker of lymphoblastoid Cells, blood and mucle cells	*−*	*−*	N
rs2097744	chr7:118382988	LSM8, ANKRD7	Response to platinum-based chemotherapy in non-small-cell lung cancer		++	++	Y
rs767441	chr15:48613619	FBN1	Breast cancer	Promoter histone marks and enhancer histone marks of adipocyte, muscle and lung cells	*−*	*−*	N
rs2104047	chr14:68287700	RAD51B	Primary biliary cirrhosis	Promoter histone marks and enhancer histone marks of blood cells, muscle cells, thymus and hematopoietic stem cells	++	++	N
rs2941742	chr6:151691853	ESR1	Bone mineral density (hip)	Promoter histone marks and enhancer histone marks of osteoblast cells, muscle cells, blood cells and liver cells	++	+	N
rs541455835 (rs67668514*)	chr17:46099939	KANSL1, MAPT	Parkinson's disease	Promoter histone marks and enhancer histone marks of brain cells, liver cells and blood cells	++	+++	Y
rs281038	chr5:156653467	SGCD	Anthropometric traits	Enhancer histone marks of foreskin melanocyte primary cells	+	*−*	Y (rs157350)
rs6990531	chr8:80483511	ZBTB10	Eating disorders	Promoter histone marks and enhancer histone marks of brain, muscle and blood cells	+	++	N
rs7697634	chr4:17965123	LCORL	Height	Promoter histone mark of liver and enhancer histone mark of pancreatic islets	++	+	Y
rs7957274	chr12:21197462	SLCO1B1	blood metabolite measurement	Promoter histone marks and enhancer histone marks of ESC and blood cells	+	++	N
rs11716984	chr3:121643560	HCLS1	neuropsychological test	Promoter histone marks and enhancer histone marks of ESC, iPSC and blood cells	++	*−*	Y
rs62288111	chr3:190946175	SNAR-I, GMNC	Alzheimers disease	Promoter histone mark of adult liver tissue and enhancer histone mark of hESC Derived CD56+ ectoderm cultured cells	+	++	N
rs34466261	chr7:104835293	LHFPL3	Obesity	Enhancer histone mark of HUES48 ESC cells	+	*−*	N
rs2149943	chr10:107811551	SORCS1	Prion diseases	Enhancer histone mark of ES-UCSF4 cells and pancreatic islets	++	+	N
rs7007731	chr8:76783496	ZFHX4	age at menarche	Enhancer histone mark of Mesenchymal cells	*−*	++	Y (rs4735738)

'*' 15 SNPs that show allelic differences in the homodimeric binding of FOXA1 to sites with DIV motifs. ‘+’ indicates luciferase expression level increased comparing to empty vector and ‘–’ that it decreased. denotes a previously used alternative

SNP ID

We became particularly interested in SNP rs2941742 located within an intron region of the *ESR1* gene (Figure 6C). This SNP maps to a region with the enhancer mark H3K27ac and the promoter mark H3K4me3 in osteoblasts, as well as H3K27ac marks in muscle and bone marrow cells (Figure 6C). The SNP rs2941742 is in LD with rs2941740 (*r*^2^ = 0.98 in the European population) linked to aberrant bone mineral density (BMD)—a trait used in the clinic to diagnose osteoporosis and the estimation of fracture risk (69). Interestingly, *ESR1* is a gene relevant for bone metabolism as osteoporosis mainly affects post-menopausal women with depressed levels of its activating ligand estrogen (70). EMSAs showed that the minor allele reduced the homodimer cooperativity of FOXA1 to the rs2941742 locus 20-fold whilst monomeric binding is not affected (Figure 6D and E). Moreover, luciferase assays revealed reduced reporter activity for the minor as compared to the major allele that is dependent on exogenous FOXA1 addition (Figure 6F). It is therefore conceivable that the perturbation of FOXA1 dimers or of related *forkhead* TFs on the rs2941742 locus modifies *ESR1* regulation and contributes to the etiology of osteoporosis.

rs5414555835 (also annotated with ID rs67668514) maps to a locus bound by FOXA1 in various cancer cell lines (Figure 6G) and displays active histone marks in liver and brain cells (Table 1). This SNP is in LD with rs17577094 at *r*^2^ = 0.97 in the European population and maps to the chr17q21.31/*MAPT* locus (71) reported to be strongly associated with Parkinson's disease (PD) (72). *MAPT* encodes for the Tau protein whose de-regulation and aberrant folding is a major cause for the disease progression. Interestingly, rs541455835 is a GTEx eQTL associated with allele specific changes in *MAPT* expression in various tissues (Supplementary Figure S6B). Moreover, rs541455835 shows a strong difference in binding and reporter gene expression in an allele-specific and FOXA1-dependent manner (Figure 6H and I). Notably, FOXA1 and FOXA2 are critical for the function of adult dopaminergic neurons (73). For example, gene delivery of FOXA2 in a mouse model for PD protected midbrain dopaminergic neurons and alleviated motor deficits (74). Therefore, the modulation of FOXA1/2 dimerization on the *MAPT* locus could hamper the neuroprotective roles of FOXA1/2 and contribute to PD. Overall, we observed significant differences of reporter expression between major and minor allele sequences for 10 of the 15 tested SNPs (Supplementary Figure S6C), five of which are also eQTLs.

## DISCUSSION

In this study, we demonstrate that FOXA1 can form a DNA-dependent homodimer in the presence of a palindromic DNA element with overlapping half-sites. Unlike some other TFs such as SOX9 that form dimers on flexibly spaced composite elements (39), FOXA1 dimerization relies on precise half-site spacing. Interestingly, the DIV motif was also detected, but not validated, using methyl-SELEX with full length FOXA1 (33) and high-throughput SELEX (32) for members of the FOXC subfamily. Further, we noticed that in a recently reported crystal structure of DNA bound FOXO1 an arrangement of crystallographically stacked DNA helices resembling the DIV configuration (Supplementary Figure S7) (75). However, the authors did not test cooperative dimer formation on such an element. Lastly, ChIP-exonuclease sequencing studies indicated the presence of various forms of composite *forkhead* elements. First, two clustered forkhead sites (termed mesas) resembling a widely spaced CON element (Figure 1A) were reported (31). Second, a DIV signature was seen in glucocorticoid receptor (GR) ChIP-exo data showing signatures of cooperative binding and implying roles of the DIV for GR recruitment to chromatin (30). Collectively, the DIV motif could be a broadly used *forkhead* recognition sequence relevant for TFs beyond FOXA1. As a consequence, the dimer-modifying GWAS SNPs reported here could elicit their phenotypic consequence by perturbing regulatory programs of any *forkhead* protein. SNPs rs2941742 and rs541455835 are the most interesting candidates for a *forkhead* associated disease mechanism. They localize to distal enhancer of *ESR1* and *MAPT* genes, which are related to osteoporosis or Parkinson's disease, respectively. The rs2941742[G] osteoporosis risk allele leads to a near 100-fold decrease in FOXA1 homodimer cooperativity, and causes depressed reporter gene expression. Whilst FOXA1, to our knowledge, has not been implicated in osteoporosis, the FOXO group plays an important role in bone metabolism by regulating the redox balance, protein synthesis and differentiation in the osteoblast lineage (reviewed in (76)). Similarly, rs541455835 risk alleles promote modulation in gene expression through perturbation of FOXA1 homodimerisation. Several studies found that the original GWAS SNPs linked to the dimer modifying SNPs (rs2941740 for rs2941742; rs17577094 for rs67668514) were associated with eQTLs (77,78). Importantly, rs17577094 not only affects *MAPT* gene expression in the brain, but also in other tissues including the breast where the rs67668514 locus shows strong FOXA1 ChIP-seq signals. We also found that rs767441[C], which is one of the breast cancer risk-associated SNPs reported by Cowper-Sallari *et al* (26), destroyed dimeric binding without changing the affinity for monomeric binding. Genome editing studies provide the means to test whether perturbing *forkhead* DBD dimerization on disease-associated loci influences disease progression. Collectively, the identification of disease associated SNPs at regulatory genomic regions that reduce FOXA1 dimerization and perturb reporter gene expression corroborates our hypothesis that FOXA1 dimerization is critical for its regulatory function and contributes to disease progression.

While we showed that FOXA1/DIV configurations are linked to highly expressed genes, these binding events do not appear to act as simple transcriptional amplifier. Accordingly, the effects of minor alleles of the studied SNPs are diverse. Reporter assays showed that the presence of minor alleles could lead to elevated, reduced or unchanged expression levels relatively to major alleles (Figure 6E, F and I). Likewise, when the expression of eQTLs is compared across tissues, the minor allele can be associated with increased expression levels in one tissue but with reduced expression levels in another (Supplementary Figure S6B). This implicates that regulatory outcomes of FOXA1/DIV complexes are dependent on the overall sequence as well as the cellular context. For example, whether a FOXA1/DIV complex recruits co-activators or repressors could be influenced by peripheral sequences and change from one cell to another. The association of DIV sequences with open-close dynamics of chromatin suggests a role of FOXA1/DIV complex in chromatin remodeling. An important question is whether the regulatory consequence of FOXA1 dimers on DIV motifs is different than other configurations such as monomers, heterodimers or homodimers on CON sequences. Clearly, we identified enhancers that rely on FOXA1/DIV dimers for their activity. An intriguing possibility is that the DIV motif not only affects the strength and dynamics of binding but also has qualitative effects. A possible mechanism is that only when bound to DIV sequences FOXA1 would recruit a set of co-factors that in turn may trigger transactivation, chromatin looping or the setting of epigenetic marks in a cell type dependent manner. By contrast, alternative FOXA1 configurations induced by other DNA motifs could lead to disparate nuclear processes. Such a mechanism could explain the context specific activities of master TFs such as FOXA1 allowing them to regulate different sets of genes in a multitude of cells and at different development stages. Collectively, homodimeric FOXA1 critically contributes to its genomic binding landscape and its regulatory activity, likely by influencing chromatin dynamics and by modulating its interactome.

## Supplementary Material

Supplementary Data
