# Supplementary material for: DNA-mediated dimerization on a compact sequence signature controls enhancer engagement and regulation by FOXA1
Source: Nucleic Acids Res. 2018 Apr 14;46(11):5470–86. doi: 10.1093/nar/gky259 (PMC6009666; doi:10.1093/nar/gky259)
Supplement: Supplementary Data [file gky259_supplemental_files.pdf]

Supplementary Figure S1

A. Modelling strategy on the basis of changing H-bonding patterns of Asn165 with adenines A3'-A5' in the half-sites of the DIV (D0)

```
                                               N165 N165
                                               5'4'3'2'1'
                                                  12345
  T     ATGAGCGGCACTCCCTCCCTTTACATAGGACTACTTGGTAAATATTTACTGAATGAAGGAAAGCTGAAGTATCTG
  M1   GATGAGCGGCACTCCCTCCCTTTACATAGGACTACTTGGTAAATATTTACTGAATGAAGGAAAGCTGAAGTATCTGG
  M2   GATGAGCGGCACTCCCTCCCTTTACATAGGACTACTTGGTAAATATTTACTGAATGAAGGAAAGCTGAAGTATCTGG
  M3   GATGAGCGGCACTCCCTCCCTTTACATAGGACTACTTGGTAAATATTTACTGAATGAAGGAAAGCTGAAGTATCTGG
  M4   GATGAGCGGCACTCCCTCCCTTTACATAGGACTACTTGGTAAATATTTACTGAATGAAGGAAAGCTGAAGTATCTGG
  M5   GATGAGCGGCACTCCCTCCCTTTACATAGGACTACTTGGTAAATATTTACTGAATGAAGGAAAGCTGAAGTATCTGG
  M6   GATGAGCGGCACTCCCTCCCTTTACATAGGACTACTTGGTAAATATTTACTGAATGAAGGAAAGCTGAAGTATCTGG
  M7   GATGAGCGGCACTCCCTCCCTTTACATAGGACTACTTGGTAAATATTTACTGAATGAAGGAAAGCTGAAGTATCTGG
  M8   GATGAGCGGCACTCCCTCCCTTTACATAGGACTACTTGGTAAATATTTACTGAATGAAGGAAAGCTGAAGTATCTGG
  M9   GATGAGCGGCACTCCCTCCCTTTACATAGGACTACTTGGTAAATATTTACTGAATGAAGGAAAGCTGAAGTATCTGG
```

Symmetric (M1, M4, M7)

1VTN (PDB ID)

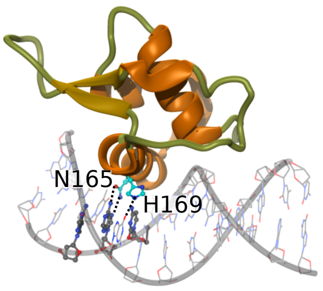

B. Superposition strategy

```
                      5' GACTAAGTCAA-C-C 3'                                       (1VTN)
5'CCGCCGCCGCCGCCGCCGCCGCCGACTAAGTCAA-C-G-TTGACTTAGTCCGCCGCCGCCGCCGCCGC 3'   (Ideal B-DNA)
3'GGCGGCGGCGGCGGCGGCGGCGGCTGATTCAGTT-G-C-AACTGAATCAGGCGGCGGCGGCGGCGGCG 5'
                                    3'C-C-AACTGAATCAG 5'                    (1VTN)


Extension:                5' GACTAAGTCAA-C-G-TTGACTTAGTC 3'        (Model from superposition)

Fragments:
5'gATGAGCGGCACTCCCTCCCTTTACATAGGACTACg 3'              5'gGTGAAGGAAAGCTGAAGTATCTGg 3'

Model:
   ATGAGCGGCACTCCCTCCCTTTACATAGGACTACTTGGTAAA-T-A-TTTACTGAATGAAGGAAAGCTGAAGTATCTG (PVT1 genomic)
```

C. Symmetric models where Asn165 binds identical Adenines (A3', A4' or A5') in both half-sites

M1

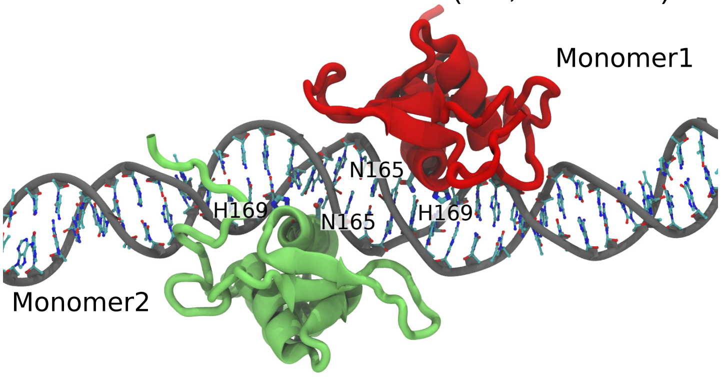

M4

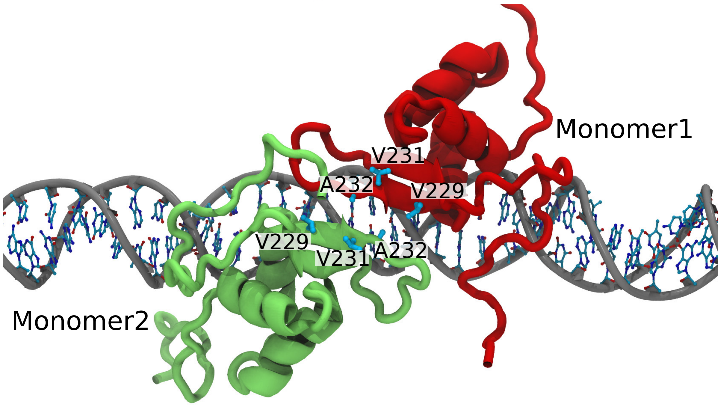

M7

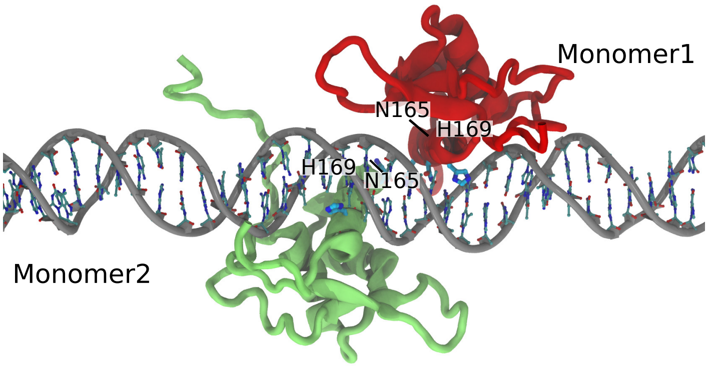

Supplementary Figure S1

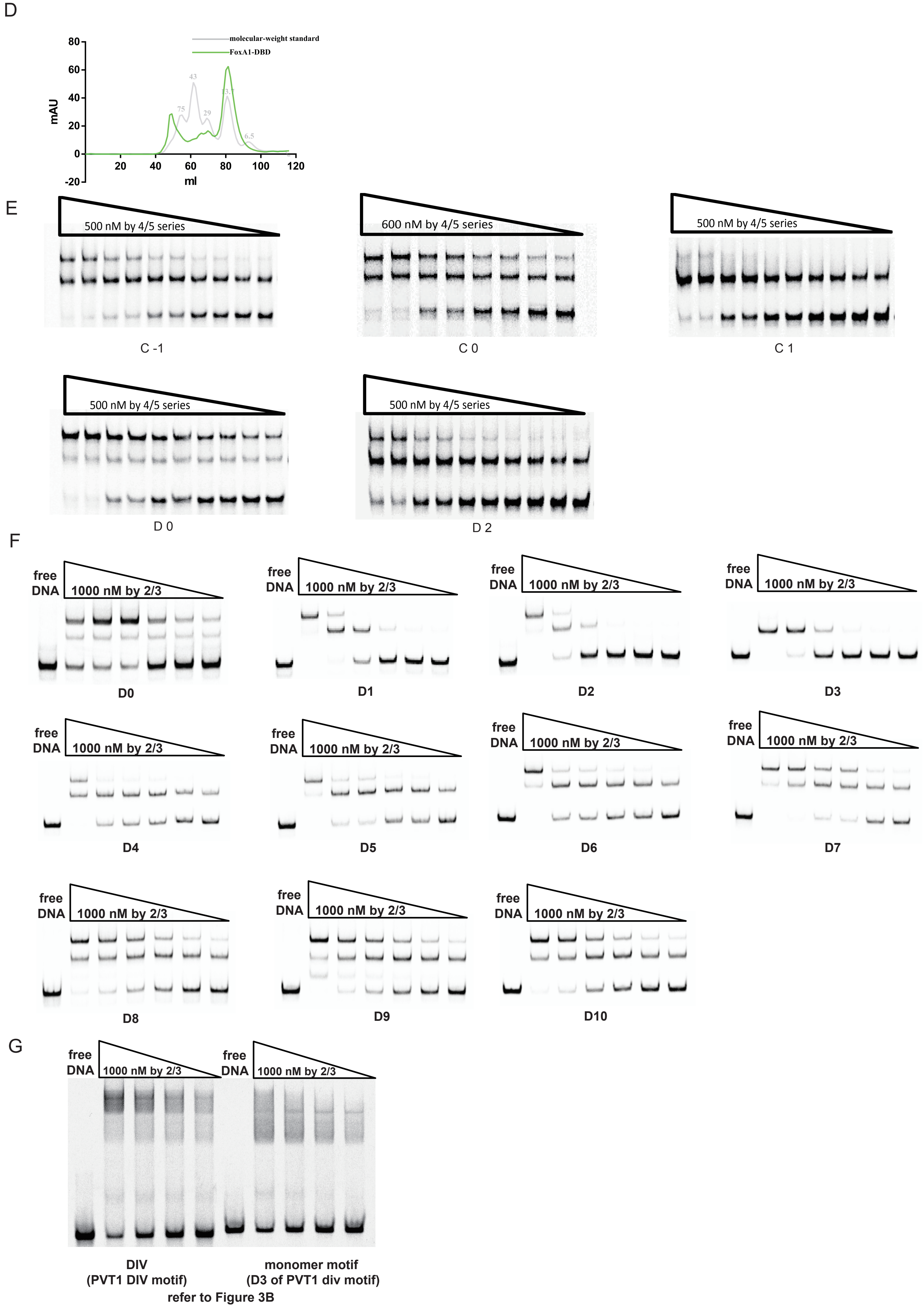

Supplementary Figure 2

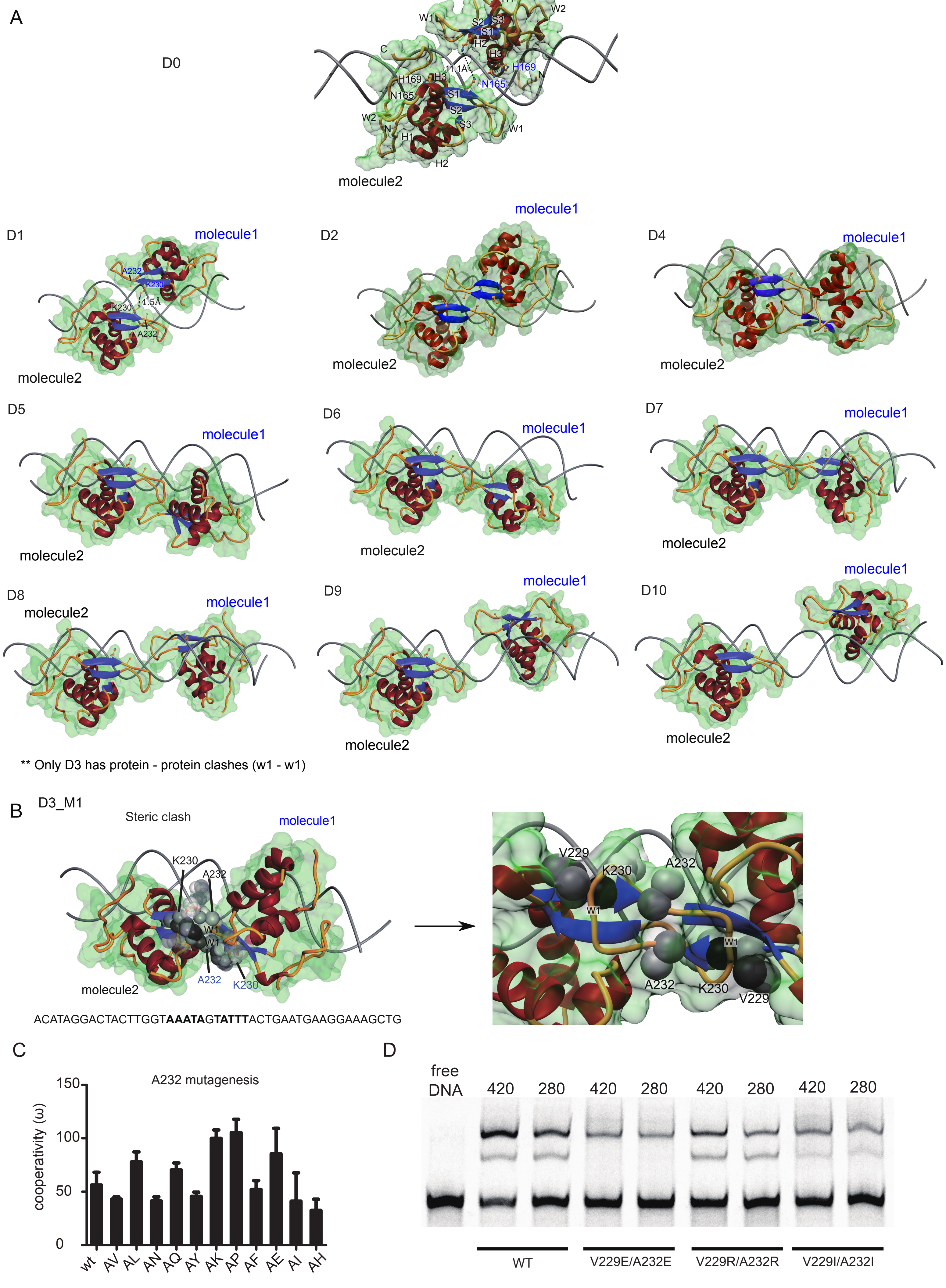

Supplementary Figure S3

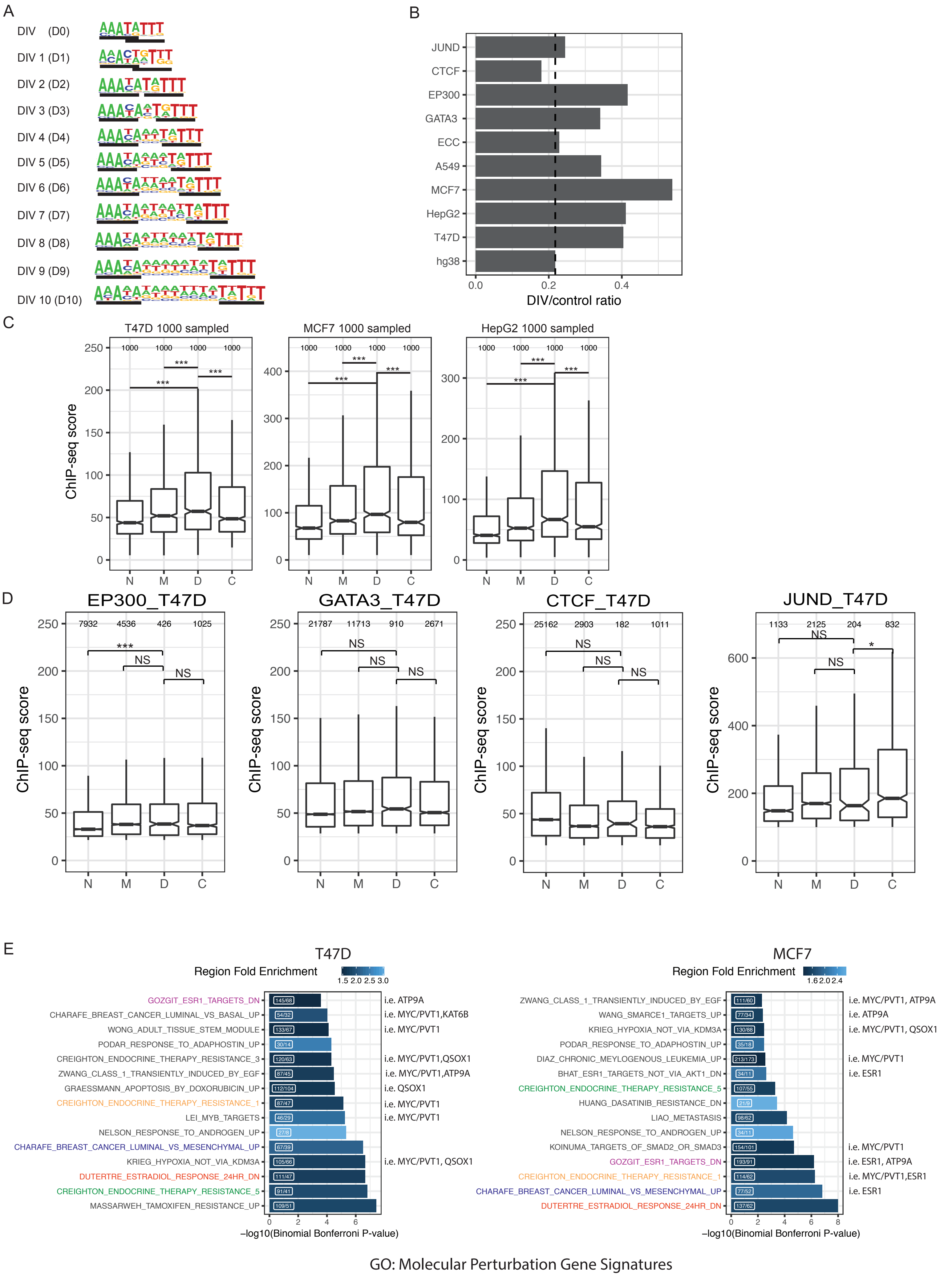

Supplementary Figure S4

A

Nearest gene	Full name	TSS to DIV motif	Role
ESR1	Estrogen Receptor 1	-32200 (intergenic)	involve in pathological processes of breast cancer (F. Holst, *et al.,* 2007)
PVT1/ MYC	Pvt1 oncogene (non-protein coding) near oncogene MYC	-20037 (intergenic)	amplification of PVT1 contributes to the pathophysiology of breast cancer (Y. Guan,W.L. Kuoet *et al.,* 2007)
ATP9A	ATPase, class II, type 9A	23641 (intron)	remodeling and intracellular transport (Takatsu *et al.,* 2011)
QSOX1	Quiescin Q6 sulfhydryl oxidase 1	2968 (intron)	associate with a highly invasive phenotype and correlates with a poor prognosis in Luminal B breast cancer (Katchman *et al.*, 2013)
KAT6B	K(lysine) acetyltransferase 6B	49717 (intron)	positive regulation on cell proliferation of prostate cancer through PI3K-AKT (W. He *et al.*, 2013)

Supplementary Figure S4B FANTOM5 expression analysis of target genes chosen for validation

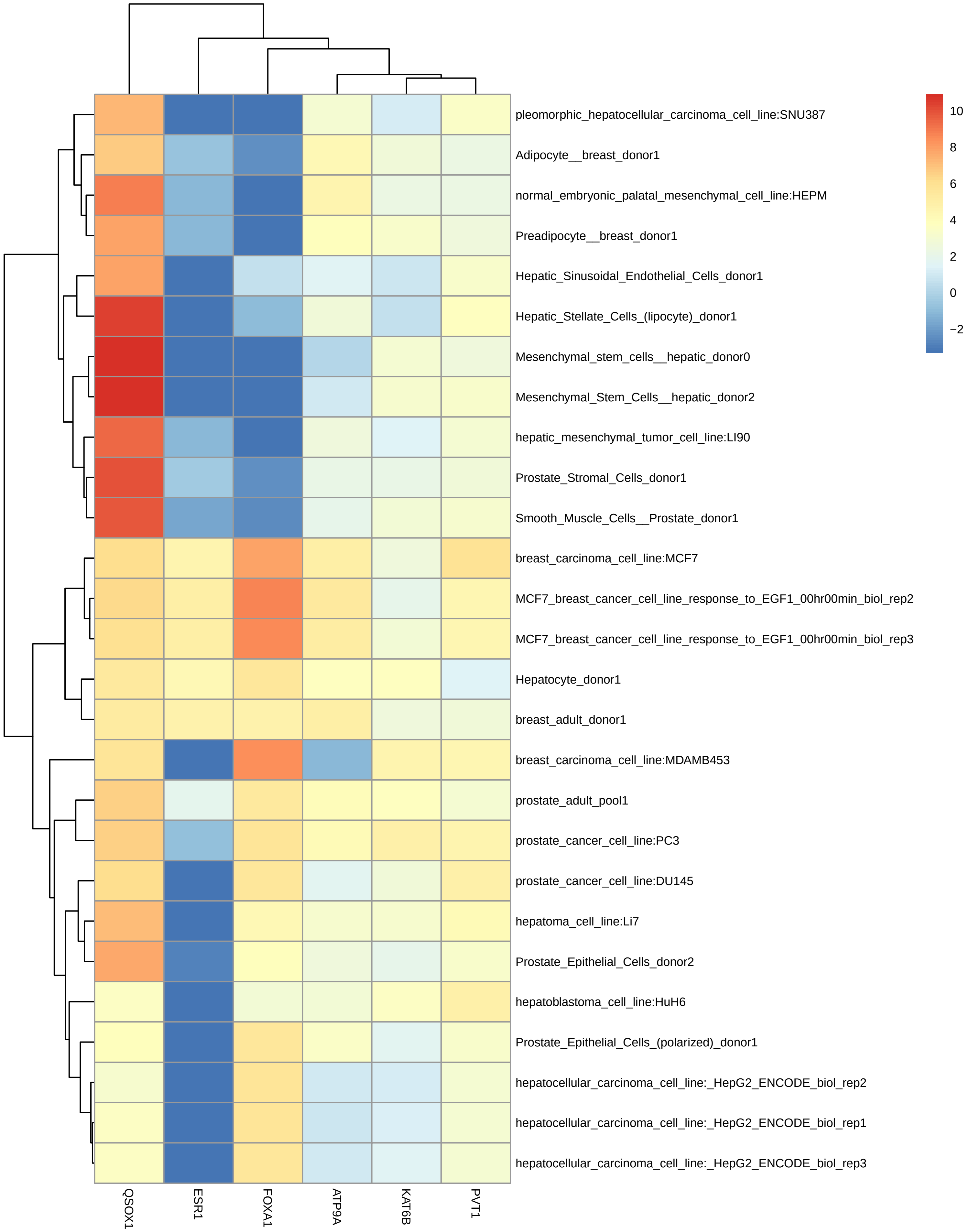

C

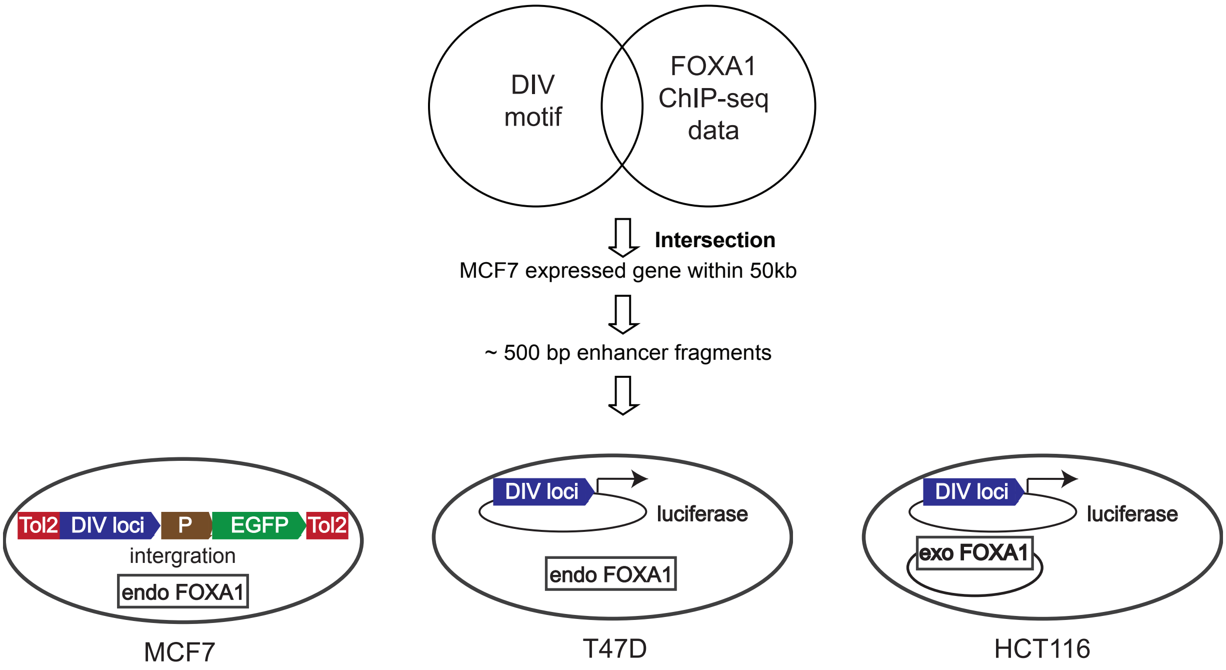
DIV
motif
FOXA1
ChIP-seq
data
Intersection
MCF7 expressed gene within 50kb
~ 500 bp enhancer fragments
Tol2
DIV loci
P
EGFP
Tol2
intergration
endo FOXA1
MCF7
DIV loci
luciferase
endo FOXA1
T47D
DIV loci
luciferase
exo FOXA1
HCT116

Supplementary Figure S4D FACS results

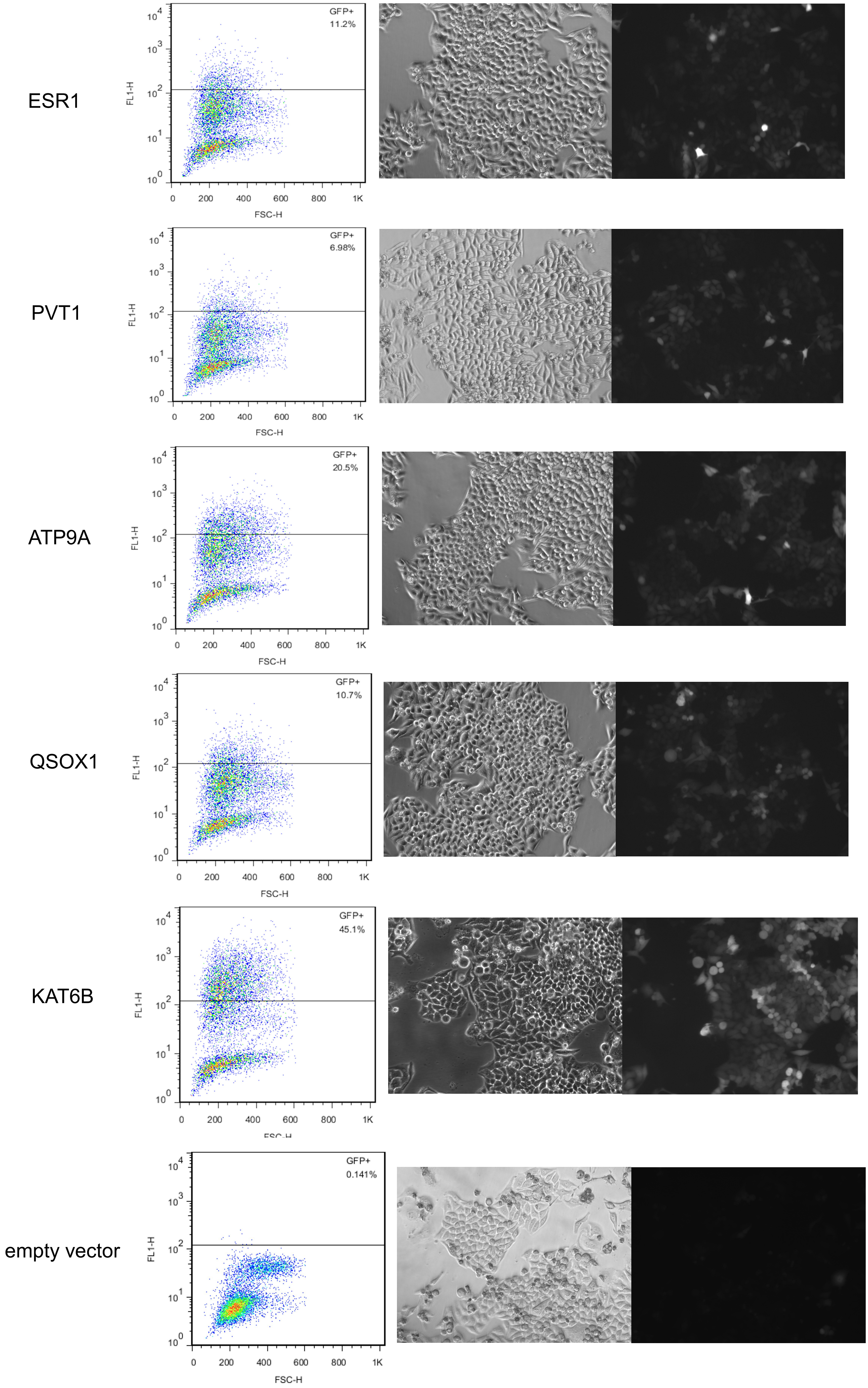

Supplementary Figure S5A

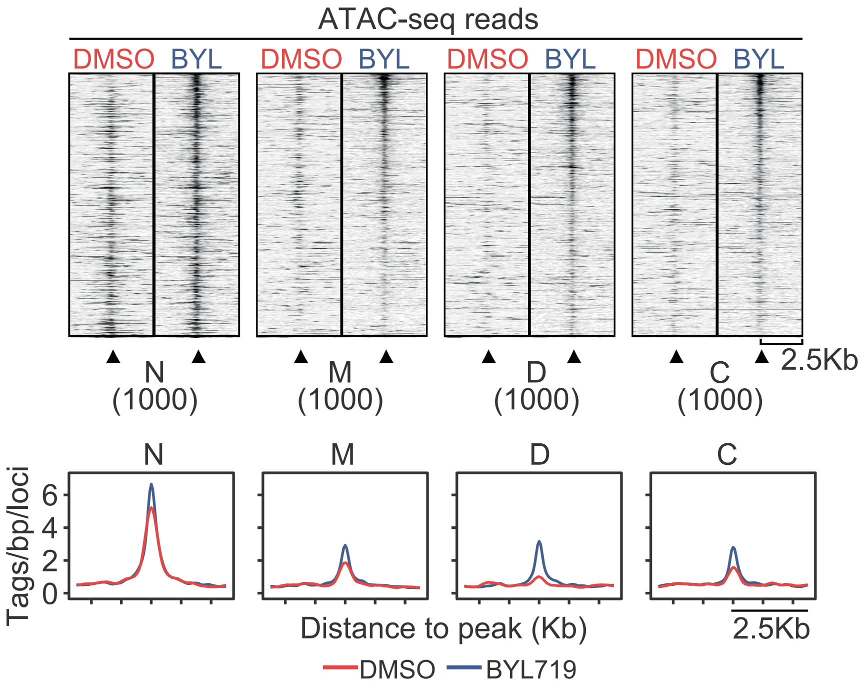

Supplementary Figure S6A

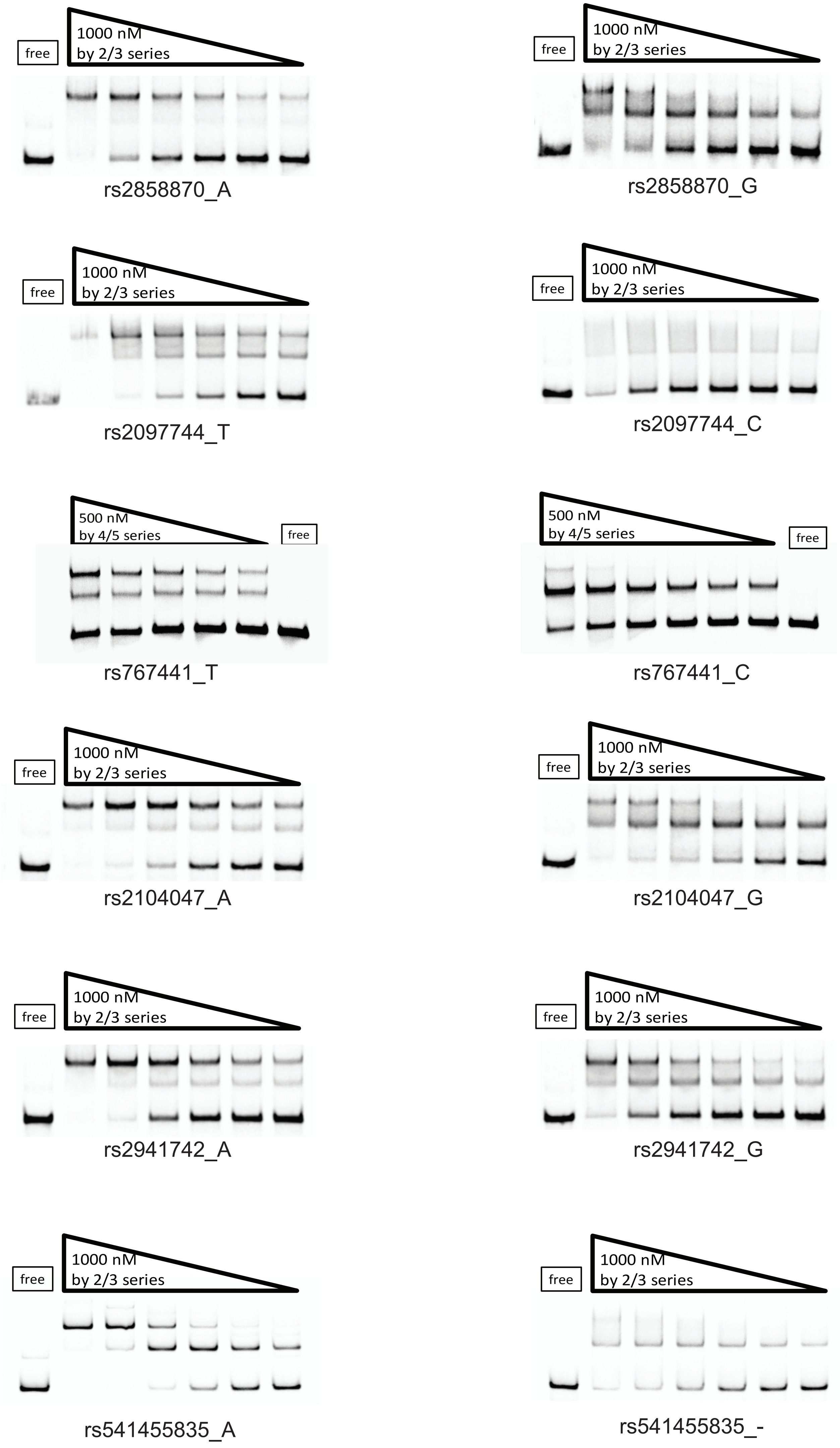

Supplementary Figure S6A

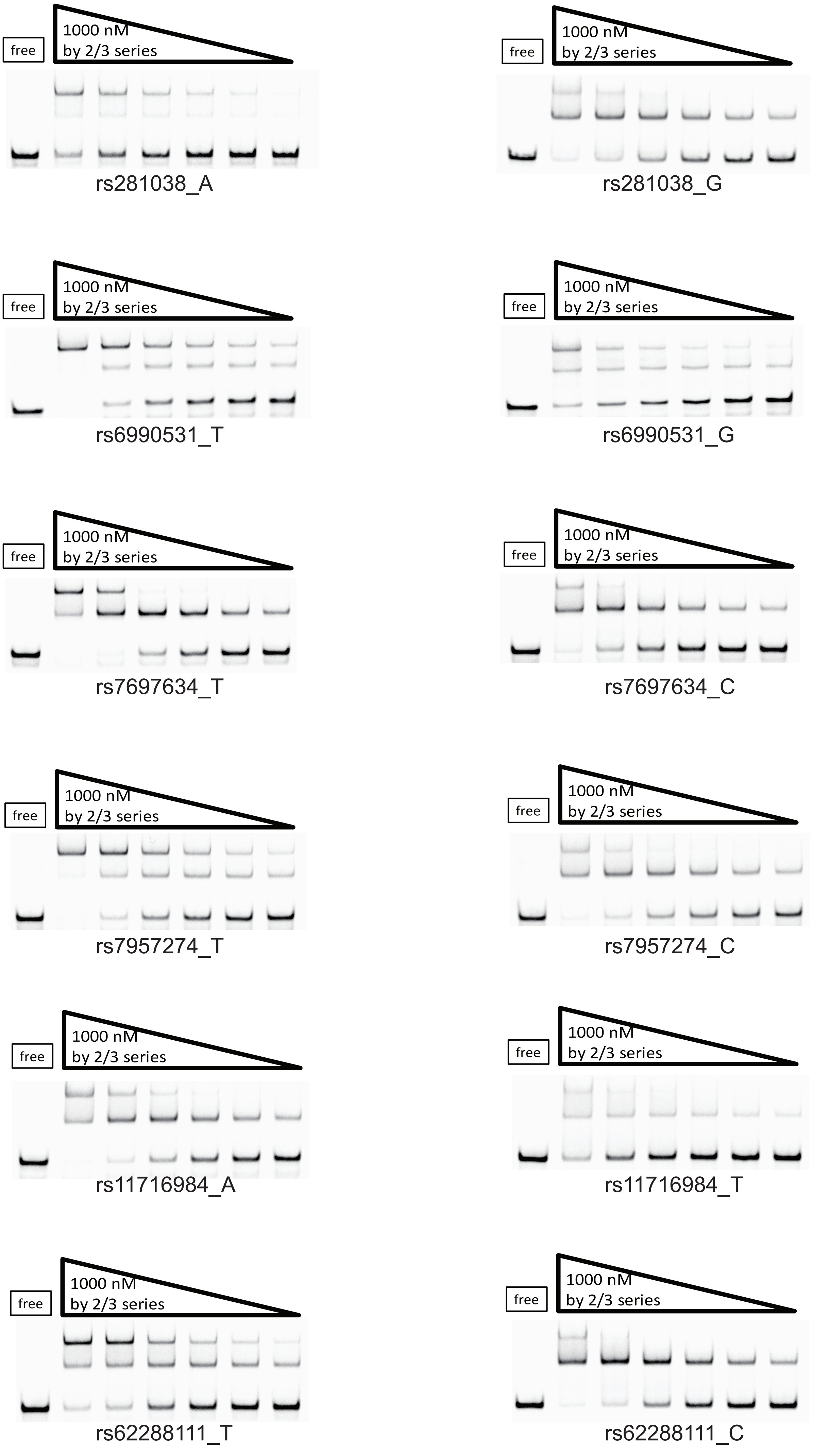

Supplementary Figure S6A

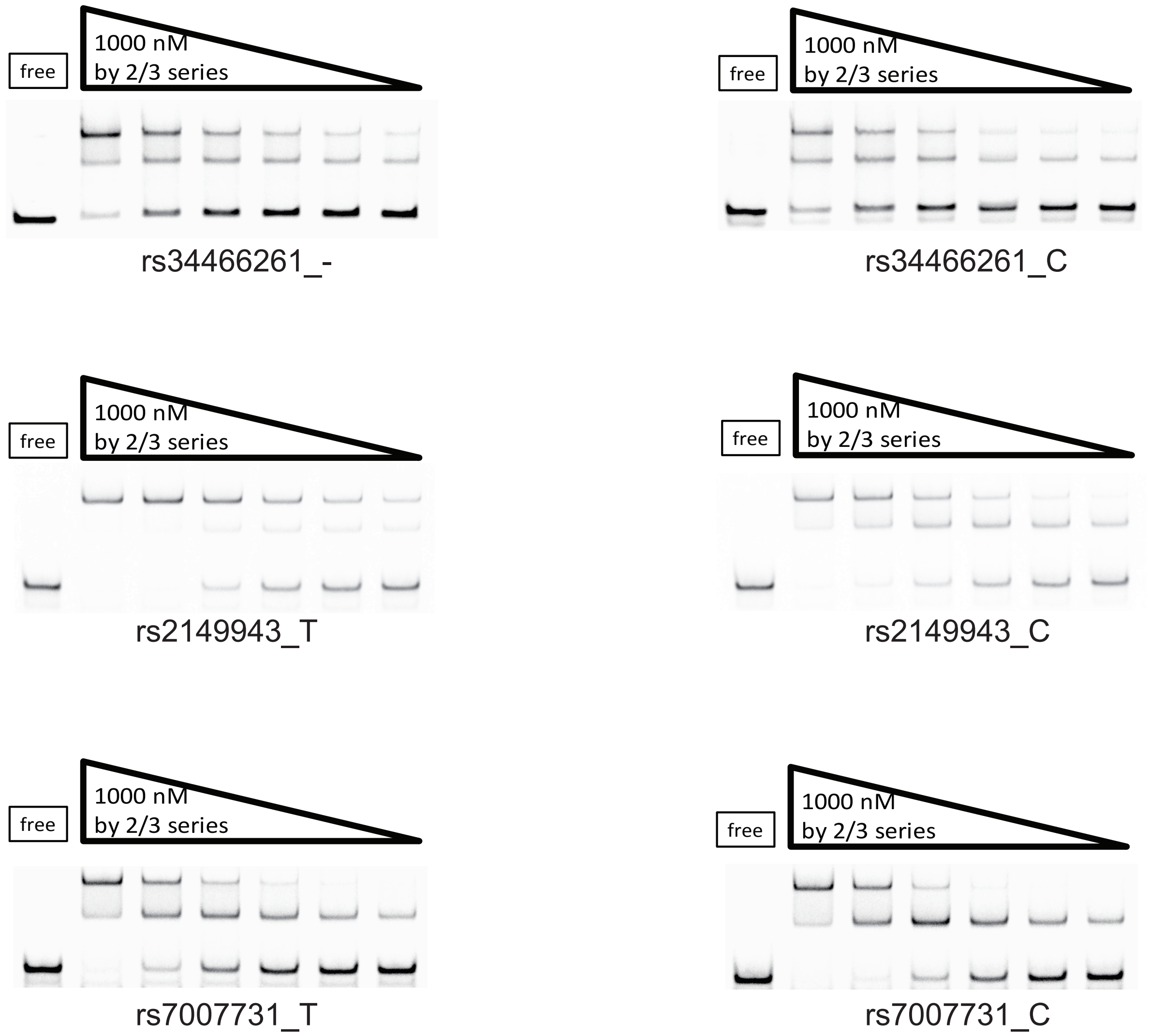

Supplementary Figure S6B

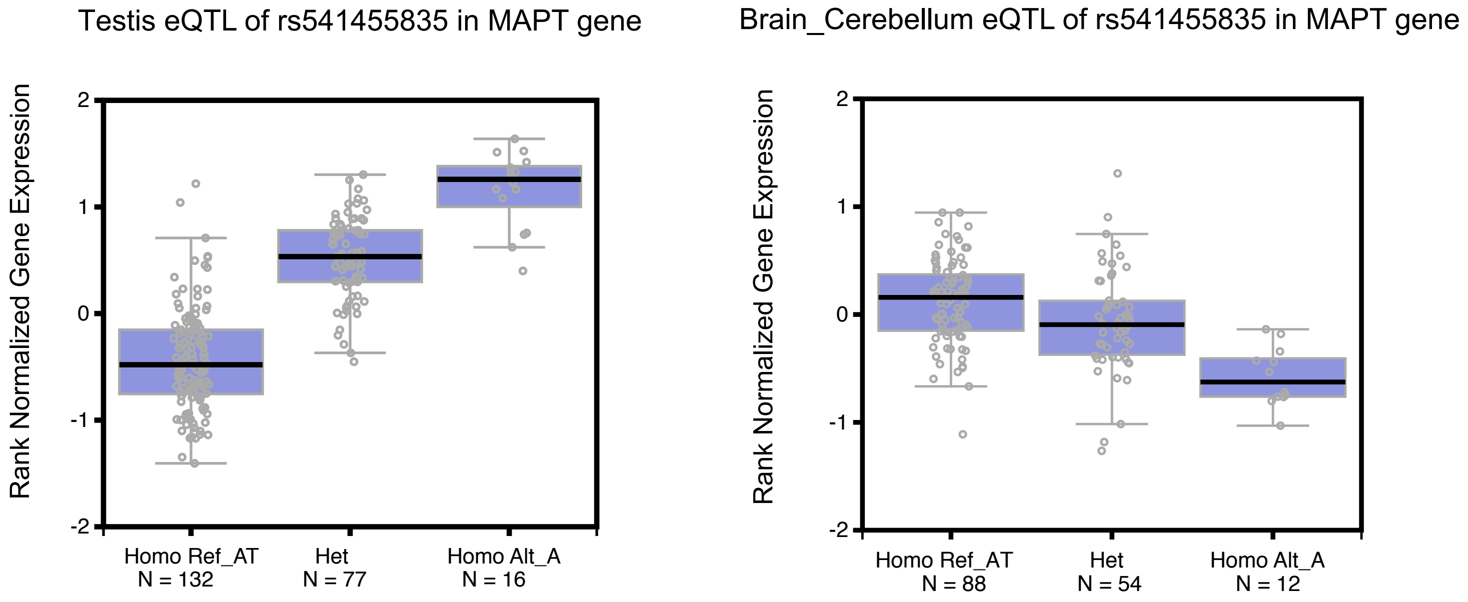

Supplementary Figure S6C

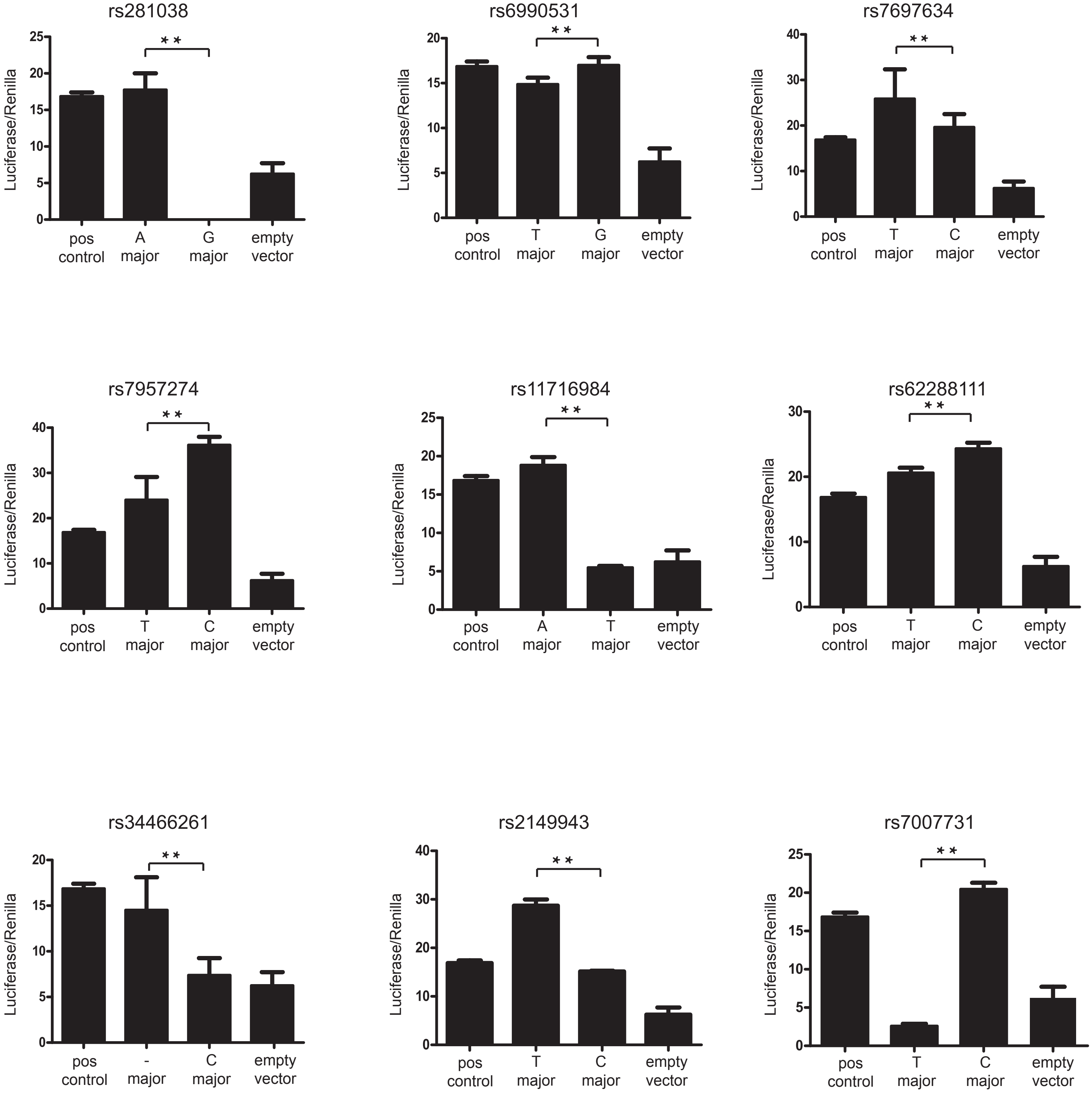

Supplementary Figure S7

A. Symmetry Mate FOXO1 (PDB ID:5DUI)

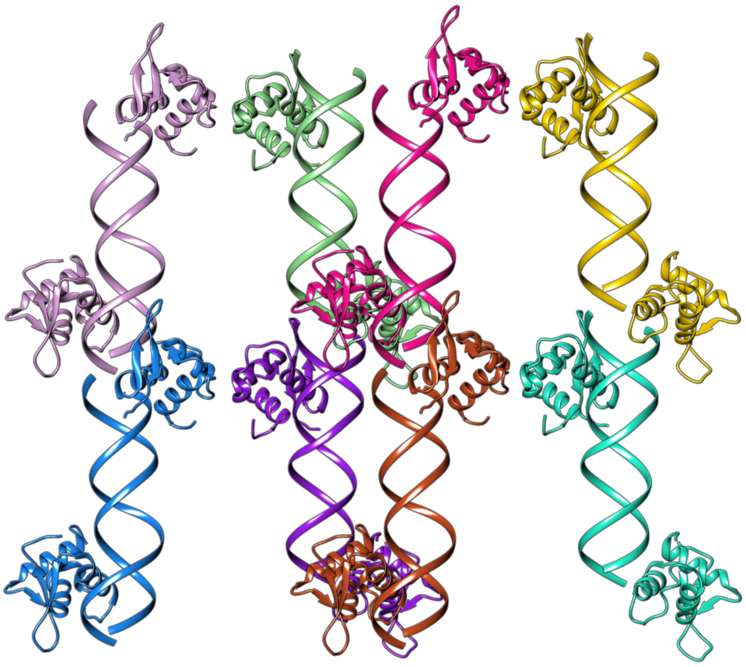

B. Superimposition of DIV on symmetry with 5DUI:

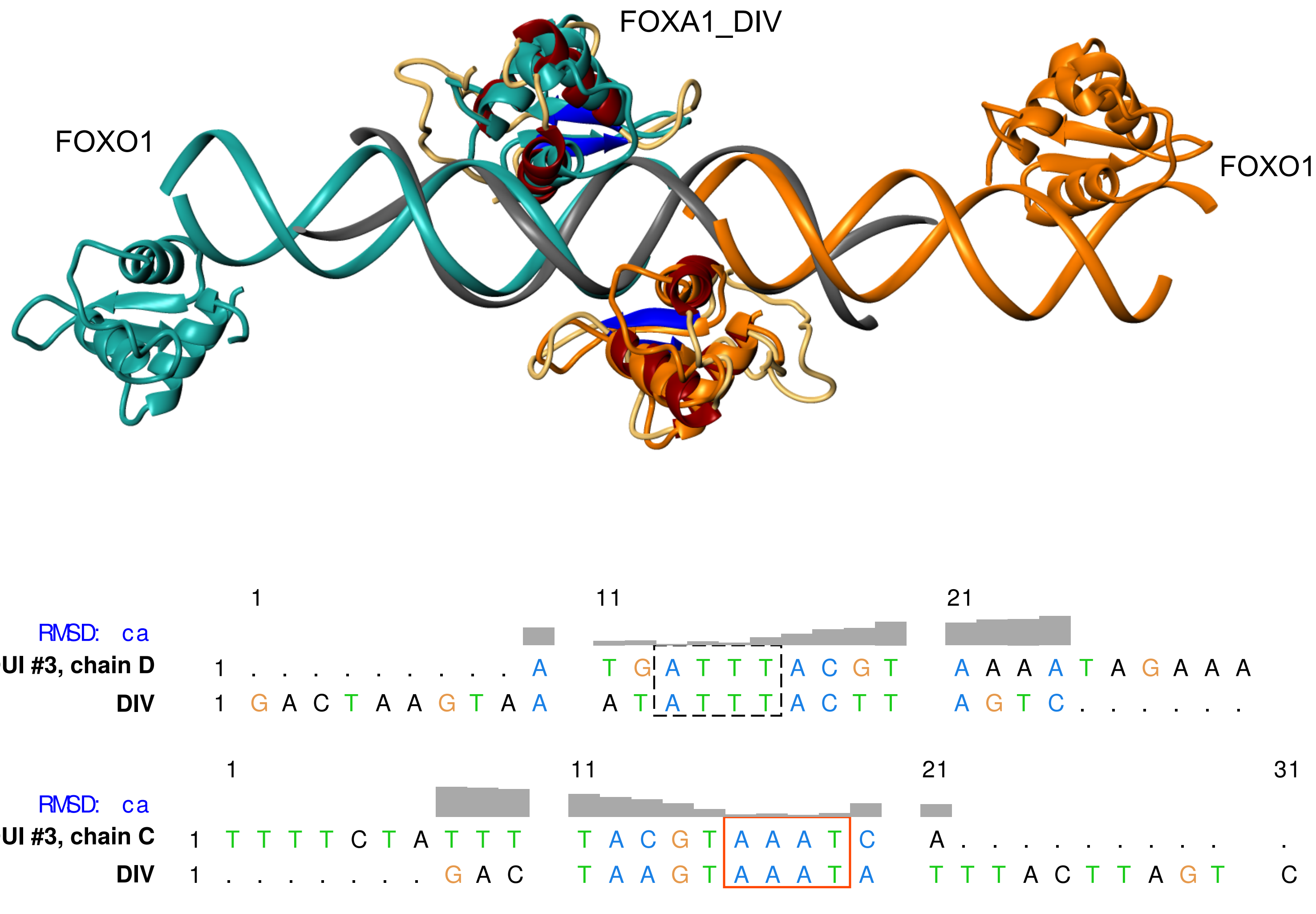

Supplementary Figure legends

Supplementary Figure S1

A. Alternative positions for the interactions of Asn165 with Adenines 3', 4' or 5' of a half-site of the DIV (D0) element. We shuffled the contacts of Asn165 with these three Adenines and created 9 models three of which are symmetric (termed D0_M1, D0_M4 and D0_M7).

B. Alignment strategy for the FOXA1 monomer models with a B-DNA template with DIV sequence. Nucleotides CAAC (yellow) are used for the superposition. Cyan marks sequences used to superimpose extending flanks. Monomer models are based on pdb-id 1VTN.

C. Cartoon of the symmetric models D0_M1, D0_M4 and D0_M7 highlighting residues nearest to the neighboring FOXA1 DBD.

D. Gel-filtration chromatogram using Superdex 75 column connected to an AktaExpress system. The green line represents the human FOXA1 DBD with a size of 16 KD, gray line shows the molecular-weight standards.

E. EMSAs using DIV (D0), CON (C0) and elements with inserted spacers.

F. EMSAs with D0 elements and elements with spacers D1 to D10.

G. EMSA using the DIV from the *PVT1* enhancer and mutated version (PVT1_monomer motif refer to Figure 4B) and full length FoxA1 protein.

Supplementary Figure S2

A and B. Structural models of FOXA1 dimers on DIV sequences with spacers 1-10 (D1 to D10). (B) The FOXA1/D3 model is represented in more detail to highlight molecular clashes.

C. Cooperativity values (ω) calculated from EMSAs of FOXA1 DBDs with a range of mutations to Ala232.

D. EMSA with 100 nM cy5 labeled DIV DNA and 420 nM or 280 nM of FOXA1 DBD

WT proteins and in comparison to the double mutants V229E/A232E; V229R/A232R and V229I/A232I.

Supplementary Figure S3

A. Sequence logos of control DIV elements with spacers 1-10 generated with HOMER.

B. Ratios of of DIV/control motif counts in the human genome hg38 and after intersection with ChIP-seq peaks.

C. Boxplots of ChIP-seq scores (narrowpeak signal values) for 1000 randomly sampled peaks from the four peak categories. *p*-values are calculated using pairwise comparisons with the unpaired Wilcoxon rank sum test (R function pairwise.wilcox.test) and adjusted using the Holm method. *** indicated p-value < 0.001. Related to Figure 2C.

D. ENCODE ChIP-seq signals for EP300, GATA3, CTCF and JUND over the four binding site categories.

E. Gene ontology analysis performed using GREAT (http://great.stanford.edu) (81) and annotated gene expression signatures from genetic or chemical perturbation experiments from the Molecular Signature database (MSigDB v6.1. http://software.broadinstitute.org/gsea/index.jsp). IDs of the top 15 most enriched gene sets ranked by Binomial Bonferroni corrected p-values are given for FOXA1/DIV locations in T47D and MCF7 cells. Bars are shaded by the binomial fold enrichment of bound genomic regions in the test set over expected and numbers in the bars are observed/expected numbers of bound genes (hypergeometric). Memberships to enriched gene sets of genes selected for further analysis are indicated. Selected gene set ID's recurring in MCF7 and T47D datasets are colored.

Supplementary Figure S4

A. Five DIV loci and associated genes with potential roles in oncogenesis selected for functional analysis.

B. FANTOM5 gene expression of FOXA1 and genes chosen for validation in selected cell and tissues types.

C. Scheme for the design of the reporter assay using endogenous or exogenous FOXA1 leading to genome integration or plasmid-based readouts.

D. Representative FACS plots of the Tol2 reporter assay. The left panel shows the GFP fluorescence channel (FL-1, y-axis) and FSC (cell size, x-axis). The GFP+ value on the upper right corner shows the percentage of GFP positive cells out of 10,000 life cells. Gating was done based on the empty vector. The central panel shows phase contrast photographs of MCF7 cells and the right panel shows the same area imaged using the GFP channel.

Supplementary Figure S5

A. Heatmap of ATAC-seq reads over FOXA1 binding sites classified by binding category (Figure 3B) after treatment with DMSO or BYL719. 1000 randomly sampled sites were from each category were used for the analysis. The lower panels are aggregate pileups of ATAC-seq signals.

Supplementary Figure S6

A. EMSAs for a total of 15 diseases associated SNPs mapping to DIV elements to compare allelic dimerization differences.

B. Example showing that rs541455835 is a GTEx eQTL. The expression of the *MAPT* gene shows allele specific expression Testis.

C. Dual Luciferase assay of 9 SNPs with allele-specific expression differences.

Supplementary Figure S7

A. Packing of FOXO1 DBDs in the crystal lattice with differently colored symmetry mates (PDB ID:5DUI).

B. Superimposition of FOXA1/DIV models onto FOXO1 showing a FOXO1/DIV configuration along a crystallographic symmetry axis.

Supplementary Table 1

Primers to construct luciferase reporters

Name	Forward	Reverse
rs67668514_Luc	CGGGGTACCCCGCCTGATGCTTGTTTCTGTGC	TGTCTGTCGATATCGCTCAAAAGCAGTGTGAAACA
rs2941742_Luc	CGGGGTACCCCGGGCAATGGTTCCTGCTTAAA	TGTCTGTCGATATCCACGTATCTCCAAATGTCCAAG
rs2097744_Luc	CGGGGTACCCCGTCAACAATGGGAGCTTTTCC	TGTCTGTCGATATCGTGGGGACACAGAACCAAAC
rs2104047_Luc	CGGGGTACCCCGTCCTGGCTTCAGACCTAATCA	TGTCTGTCGATATCAGGGCTTTGGAATCAGACCT
rs7697634_Luc	CGGGGTACCCCGTTCCTATGCCAAGCGTAACA	TGTCTGTCGATATCTGGGGAGAGTGAAAATCTGG
rs281038_Luc	CGGGGTACCCCGCATTCTTTTCGGGGTTTCAA	TGTCTGTCGATATCGGCCCAGTGCTAAAGTCAGA
rs34466261_Luc	CGGGGTACCCCGGCAGTTTAACCTCCAGTGTGG	TGTCTGTCGATATCTTTGCCATCTCCTAGAACTGTG
rs6990531_Luc	CGGGGTACCCCGTCATTCATTCCTAATTAGTCCATGTT	TGTCTGTCGATATCCGGATCATGAGGTCTGGAGT
rs2149943_Luc	CGGGGTACCCCGGGATCAAGAACAGCTGCACA	TGTCTGTCGATATCGATCAGCGTGGTAAAGCAAA
rs767441_Luc	CGGGGTACCCCGTGTCACAATTCATGAAGCCATA	TGTCTGTCGATATCGCAGTGGTGCGATGTCAG
rs62288111_Luc	CGGGGTACCCCGTGATAAGAAATGGCCCAGGT	TGTCTGTCGATATCTGCTAAGCTGAGGAATGAAGC
rs11716984_Luc	CGGGGTACCCCGGCATGCCTGCCTTATCTACC	TGTCTGTCGATATCCTTTTGCTCACAAGCCACAA
rs7957274_Luc	CGGGGTACCCCGTGGAAACAAATGATGAAAAGGA	TGTCTGTCGATATCTCAGCATCACCCACTAGGTTC
ESR1_Luc	CGGGGTACCCCGGATTTTAGCCATCCAAGAAAGG	GGAAGATCTTCCCTCAGGTGTGTGGAGGGATT
ATP9A_Luc	CGGGGTACCCCGGCCTCAGTCTTCCAAAGTGG	GGAAGATCTTCCCTGGGACTACAGCCACACCT
KAT6B_Luc	CGGGGTACCCCGCCTTTCAAGGTCAGGGGAGA	GGAAGATCTTCCCCCAGAAACCTTCTGACTGG
PVT1_Luc	CGGGGTACCCCGCTGGTGGACATTGGGTCTTT	CCGCTCGAGCGGATCATTGCCTTGACCCAGAC
QSOX1_Luc	CGGGGTACCCCGGGTTGGTCTCGCTTCATCTC	GGAAGATCTTCCATTTTCCCGGGTAAGGATTG

Primers to construct Tol2 reporter

Name	Forward	Reverse
ESR1_Tol2	ACGCGTCGACGTCGGCCATAGCGGCCGCGGAAGATTTT AGCCATCCAAGAAAGG	CGCGGATCCGCGCTCAGGTGTGTGGAGGGATT
ATP9A_Tol2	ACGCGTCGACGTCGGCCATAGCGGCCGCGGAAGCCTCA GTCTTCCAAAGTGG	CGCGGATCCGCGCTGGGACTACAGCCACACCT
KAT6B_Tol2	ACGCGTCGACGTCGGCCATAGCGGCCGCGGAACCTTTC AAGGTCAGGGGAGA	CGCGGATCCGCGCCCAGAAACCTTCTGACTGG
PVT1_Tol2	ACGCGTCGACGTCGGCCATAGCGGCCGCGGAACTGGTG GACATTGGGTCTTT	CGCGGATCCGCGATCATTGCCTTGACCCAGAC
QSOX1_Tol2	ACGCGTCGACGTCGGCCATAGCGGCCGCGGAAGGTTGG TCTCGCTTCATCTC	CGCGGATCCGCGATTTTCCCGGGTAAGGATTG

Primers for SNP mutagenesis

Name	Sequence 1	Sequence 2
rs67668514_A-	GTGTCACAAAAGCAAAATTGATACTGCATTTAGGTAACC AAAAAG	CTTTTTGGTTACCTAAATGCAGTATCAATTTTGCTTTT GTGACAC
rs2941742_GA	CAGGATATTAACAAAATCAAAGAAAATATTTGCTTTTG AGAAATAGGAAGGGAT	ATCCCTTCCTATTTCTCAAAAGCAAATATTTTCTTT GATTTTGTTAATATCCTG
rs2097744_TC	CTTTTAATTTTCTGTTAAAAACCTAAGCATTTGCACAA ATAATTTATAAAATTCAGCTGTGTA	TACACAGCTGAATTTTATAAATTATTTGTGCAAATG CTTAGGTTTTTAACAGAAAATTAAAAG
rs2104047_TC	CCACTACTGACAAATAACAAGTATTTGCTGTCAATATG TTCAAAAGCAATAAC	GTTATTGCTTTTGAACATATTGACAGCAAATACTTG TTATTTGTCAGTAGTGG
rs7697634_TC	ATTCATTGCCATTTATTTGACTGTGTAAGCATTTGTTG TAATTAACATTTTCTTGAAG	CTTCAAGAAAATGTTAATTACAACAAATGCTTACAC AGTCAAATAAATGGCAATGAAT
rs281038_AG	GCTTTAGAATTACGTAAGAAAACATTTTTATGTTTTCA TGACAAAGGTGACATTACA	TGTAATGTCACCTTTGTCATGAAAACATAAAAATGT TTTCTTACGTAATTCTAAAGC
rs34466261_C---	TTAAATATCGTTCCATCAACCAAATTTGCTGTGTGTTC TTCTAGTC	GACTAGAAGAACACACAGCAAATTTGGTTGATGGAA CGATATTTAA
rs6990531_TG	TTCATTCAAAAACTGTTAAATTCAAATCTTTGCTACTT CTTATCTCGTATCCTAC	GTAGGATACGAGATAAGAAGTAGCAAAGATTTGAAT TTAACAGTTTTTGAATGAA
rs2149943_TC	CTCTCTAAATTCTATGAACTAATGTTTACTGGGCACTC AGTAAACATATT	AATATGTTTACTGAGTGCCCAGTAAACATTAGTTCA TAGAATTTAGAGAG
rs767441_TC	GTTTCCAAACTTTTGCTAAAGAAATGTTTACATGTTTT GGCCTTTATTTTTTTCTG	CAGAAAAAAATAAAGGCCAAAACATGTAAACATTTC TTTAGCAAAAGTTTGGAAAC
rs62288111_TC	CCAGTATCTTTTCTTTTATAGTATATACTTCAGAATCA ACACTTGCATTTTTTATATTTGAG	CTCAAATATAAAAAATGCAAGTGTTGATTCTGAAGT ATATACTATAAAAGAAAAGATACTGG
rs11716984_AT	TTATCCCACCAAAATCCACATATATGTGTTTCCATTAT TGAGTAATG	CATTACTCAATAATGGAAACACATATATGTGGATTT TGGTGGGATAA
rs7957274_TC	ACATCCATTTTTTATGAGCCAAATAGTAAACATTAATT TTAGTATTATATGGTCTAAACTG	CAGTTTAGACCATATAATACTAAAATTAATGTTTAC TATTTGGCTCATAAAAAATGGATGT

Primers for FOXA1 A232 mutagenesis

Name	Sequence 1	Sequence 2
A232L	CTTGTCCGGGGAGCGTAACACCTTGACGAAGCAG	CTGCTTCGTCAAGGTGTTACGCTCCCCGGACAAG
A232N	GCTTGTCCGGGGAGCGATTCACCTTGACGAAGCAG	CTGCTTCGTCAAGGTGAATCGCTCCCCGGACAAGC
A232Q	CTGCTTCGTCAAGGTGCAGCGCTCCCCGGACAAGC	GCTTGTCCGGGGAGCGCTGCACCTTGACGAAGCAG
A232Y	GCTTGTCCGGGGAGCGATACACCTTGACGAAGCAG	CTGCTTCGTCAAGGTGTATCGCTCCCCGGACAAGC
A232F	GCTTGTCCGGGGAGCGGAACACCTTGACGAAGCAG	CTGCTTCGTCAAGGTGTTCCGCTCCCCGGACAAGC
A232H	CTGCTTCGTCAAGGTGCATCGCTCCCCGGACAAGC	GCTTGTCCGGGGAGCGATGCACCTTGACGAAGCAG
A232I	CTTGTCCGGGGAGCGTATCACCTTGACGAAGCAG	CTGCTTCGTCAAGGTGATACGCTCCCCGGACAAG
A232E	GTCCGGGGAGCGTTCCACCTTGACGAA	TTCGTCAAGGTGGAACGCTCCCCGGAC
A232K	CTTGTCCGGGGAGCGTTTCACCTTGACGAAGCAG	CTGCTTCGTCAAGGTGAAACGCTCCCCGGACAAG
A232P	CCGGGGAGCGTGGCACCTTGACGAA	TTCGTCAAGGTGCCACGCTCCCCGG
A232V	GTCCGGGGAGCGTACCACCTTGACGAA	TTCGTCAAGGTGGTACGCTCCCCGGAC
V229R/A232R	CTTGTCCGGGGAGCGTCTCACCTTGCGGAAGCAGTCATT	CTTCAATGACTGCTTCCGCAAGGTGAGACGCTCCCCGG
V229E/A232E	GGGGAGCGTTCCACCTTCTCGAAGCAGTCATTGAAG	CTTCAATGACTGCTTCGAGAAGGTGGAACGCTCCCC
V229I/A232I	GGAGCGTATCACCTTGATGAAGCAGTCATTGAAGG	CCTTCAATGACTGCTTCATCAAGGTGATACGCTCC

Primers for mutagenesis of luciferase and Tol2 reporters

Name	Sequence 1	Sequence 2
ESR1_monomer	TGACTGCAGTAGGCACTCAGTAAATACTATTTATTGATGGTCCTG	CAGGACCATCAATAAATAGTATTTACTGAGTGCCTACTCAGTCA
PVT1_monomer	CTTTACATAGGACTACTTGGTAAATACTATTTACTGAATGAAGGAAAGC	GCTTTCCTTCATTCAGTAAATAGTATTTACCAAGTAGTCTATGTAAAG
QSOX1_monomer	CACTGGCTAATCAATCAGCAAATACTATTTGTTGAGTGATAAGCATTG	CAATGCTTATCACTCAACAAATAGTATTTGCTGATTGATAGCCAGTG
KAT6B_monomer	TATAAACCAGGAAGGGAACAAATACTATTTACACTTGCAACTTCATGTC	GACATGAAGTTGCAAGTGTAAATAGTATTTGTTCCCTTCTGGTTTATA
ATP9A_monomer	TCAGTTAGCACTGCGCAAATACTATTTGCTGAATTGTCTGC	GCAGACAATTCAGCAAATAGTATTTGCGCAGTGCTAACGA
ESR1_no binding	ATCATCCCATAATCAGGACCATCAATACCTCCTCACTGAGTGCCTACTGCAGTCAAGACAC	GTGTCTTGACTGCAGTAGGCACTCAGTGAGGAGGTATTATGGTCCTGATTATGGGATGAT
PVT1_no binding	CAGATACTTCAGCTTTCCTTCATTCAGTACCTCCTCACCAAGTAGTCCTATGTAAAGGGAGGGAG	CTCCCTCCCTTTACATAGGACTACTTGGTGAGGAGGTATGAATGAAGGAAAGCTGAAGTATCTG
QSOX1_no binding	TGCAATGCAAACAATGCTTATCACTCAACACCTCCTCGCTGATTGATTAGCCAGTGAATAGCAATGA	TCATTGCTATTCACTGGCTAATCAATCAGCGAGGAGGTTTGAGTGATAAGCATTGTTTGCATTGCA
KAT6B_no binding	TAGGCAATGTTGACATGAAGTTGCAAGTGTACCTCCTCGTTCCCTTCCTGGTTTATAAATTTCATGATG	CATCATGAAATTTATAAACCAGGAAGGGAACGAGGAGGACACTTGCAACTTCATGTCAACATTGCCTA
ATP9A_no binding	CCCCTATAATCAGTTAGCACTGCGCGAGGAGGTGCTGAATTGTCTGCATGAC	GGTGTGTCATGCAGACAATTCAGCACCTCCTCGCGCAGGCTAACTGATTAT

EMSA oligos (the core DIV elements are underlined in the forward sequence and the spacer is in bold)

Name	Forward	Reverse
D0	CCGCCGTAAGTAAATATTTACTTAGCCGCC	GGCGGCTAAGTAAATATTTACTTACGGCGG
D2	CCGCCGTAAGTAAATATATTTACTTAGCCGCC	GGCGGCTAAGTAAATATATTTACTTACGGCGG
C0	CCGCCGTATTTACTGAGTAAATAGCCGCC	GGCGGCTATTTACTCAGTAAATACGGCGG
C1	CCGCCGTATTTACTTAAGTAAATAGCCGCC	GGCGGCTATTTACTTAAGTAAATACGGCGG
C-1	CCGCCGTATTTACTAGTAAATAGCCGCC	GGCGGCTATTTACTAGTAAATACGGCGG
DIV	CCGCCGCCGCCGTAAGTAAATATTTACTTAGCCGCCGCCC	GGGCGGCGGCTAAGTAAATATTTACTTACGGCGGCGGCGG
DIV1	CCGCCGCCGCCGTAAGTAAA**C**TATTTACTTAGCCGCCGCC	GGCGGCGGCTAAGTAAATAGTTTACTTACGGCGGCGGCGG
DIV2	CCCGCCGCCGTAAGTAAATA**TA**TTTACTTAGCCGCCGCCC	GGGCGGCGGCTAAGTAAATATATTTACTTACGGCGGCGGG
DIV3	CCGCCGCCGTAAGTAAATA**CTA**TTTACTTAGCCGCCGCCC	GGGCGGCGGCTAAGTAAATAGTATTTACTTACGGCGGCGG
DIV4	CCGCCGCCGTAAGTAAA**TATA**TATTTACTTAGCCGCCGCC	GGCGGCGGCTAAGTAAATATATATTTACTTACGGCGGCGG
DIV5	GCCCGCCGTAAGTAAA**TAGAC**TATTTACTTAGCCGCCGCC	GGCGGCGGCTAAGTAAATAGTCTATTTACTTACGGCGGGC
DIV6	GCCCGCCGTAAGTAAA**TAGACG**TATTTACTTAGCCGCCGC	GCGGCGGCTAAGTAAATACGTCTATTTACTTACGGCGGGC
DIV7	CCCGCCGTAAGTAAA**TAGACGA**TATTTACTTAGCCGCCGC	GCGGCGGCTAAGTAAATATCGTCTATTTACTTACGGCGGG
DIV8	CCCGCCGTAAGTAAA**TAGACTGA**TATTTACTTAGCCGCCC	GGGCGGCTAAGTAAATATCAGTCTATTTACTTACGGCGGG
DIV9	CCGCCGTAAGTAAA**TAGACTGAC**TATTTACTTAGCCGCCC	GGGCGGCTAAGTAAATAGTCAGTCTATTTACTTACGGCGG
DIV10	CCGCCGTAAGTAAA**TAGACTGACG**TATTTACTTAGCCGCC	GGCGGCTAAGTAAATACGTCAGTCTATTTACTTACGGCGG
ESR1_EMSA	CCGCCGTATCTCTAAATATTTTCTTTCAGCCGCC	GGCGGCTGAAAGAAAATATTTAGAGATACGGCGG
ATP9A_EMSA	CCGCCGACTGCGCAAATATTTGCTGAATGCCGCC	GGCGGCATTCAGCAAATATTTGCGCAGTCGGCGG
KAT6B_EMSA	CCGCCGAGGGAACAAATATTTACACTTGGCCGCC	GGCGGCCAAGTGTAAATATTTGTTCCCTCGGCGG
PVT1_EMSA	CCGCCGACTTGGTAAATATTTACTGAATGCCGCC	GGCGGCATTCAGTAAATATTTACCAAGTCGGCGG
QSOX1_EMSA	CCGCCGAATCAGCAAATATTTGTTGAGTGCCGCC	GGCGGCACTCAACAAATATTTGCTGATTCGGCGG
rs67668514_A_EMSA	CCGCCGGTATCAATATTTGCTTTTGCCGCC	GGCGGCAAAAGCAAATATTGATACCGGCGG
rs67668514_-_EMSA	CCGCCGGTATCAATTTTGCTTTTGGCCGCC	GGCGGCCAAAAGCAAAATTGATACCGGCGG

rs2941742_A_EMSA	CCGCCGAAGCAAATATTTTCTTTGGCCGCC	GGCGGCCAAAGAAAATATTTGCTTCGGCGG
rs2941742_G_EMSA	CCGCCGAAGCAAATGTTTTCTTTGGCCGCC	GGCGGCCAAAGAAAACATTTGCTTCGGCGG
rs2097744_T_EMSA	CCGCCGGTGCAAATGTTTAGGTTTGCCGCC	GGCGGCAAACCTAAACATTTGCACCGGCGG
rs2097744_C_EMSA	CCGCCGGTGCAAATGCTTAGGTTTGCCGCC	GGCGGCAAACCTAAGCATTTGCACCGGCGG
rs2104047_A_EMSA	CCGCCGTAACAAATATTTGCTGTCGCCGCC	GGCGGCGACAGCAAATATTTGTTACGGCGG
rs2104047_G_EMSA	CCGCCGTAACAAGTATTTGCTGTCGCCGCC	GGCGGCGACAGCAAATACTTGTTACGGCGG
rs7697634_C_EMSA	CCGCCGCAACAAATGCTTACACAGGCCGCC	GGCGGCCTGTGTAAGCATTTGTTGCGGCGG
rs7697634_T_EMSA	CCGCCGCAACAAATGTTTACACAGGCCGCC	GGCGGCCTGTGTAAACATTTGTTGCGGCGG
rs281038_A_EMSA	CCGCCGATAAAAATATTTTCTTACGCCGCC	GGCGGCGTAAGAAAATATTTTTATCGGCGG
rs281038_G_EMSA	CCGCCGATAAAAATGTTTTCTTACGCCGCC	GGCGGCGTAAGAAAACATTTTTATCGGCGG
rs34466261_-_EMSA	CCGCCGCAACCAAATTTGCTGTGGCCGCC	GGCGGCCACAGCAAATTTGGTTGCGGCGG
rs34466261_C_EMSA	CCGCCGCAACCAACATTTGCTGTGGCCGCC	GGCGGCCACAGCAAATGTTGGTTGCGGCGG
rs6990531_G_EMSA	CCGCCGTAGCAAAGATTTGAATTTGCCGCC	GGCGGCAAATTCAAATCTTTGCTACGGCGG
rs6990531_T_EMSA	CCGCCGTAGCAAATATTTGAATTTGCCGCC	GGCGGCAAATTCAAATATTTGCTACGGCGG
rs2149943_T_EMSA	CCGCCGCAGTAAATATTAGTTCATGCCGCC	GGCGGCATGAACTAATATTTACTGCGGCGG
rs2149943_C_EMSA	CCGCCGCAGTAAACATTAGTTCATGCCGCC	GGCGGCATGAACTAATGTTTACTGCGGCGG
rs767441_T_EMSA	CCGCCGATGTAAATATTTCTTTAGGCCGCC	GGCGGCCTAAAGAAATATTTACATCGGCGG
rs767441_C_EMSA	CCGCCGATGTAAACATTTCTTTAGGCCGCC	GGCGGCCTAAAGAAATGTTTACATCGGCGG
rs62288111_T_EMSA	CCGCCGAGAATCAATACTTGCATGCCGCC	GGCGGCATGCAAGTATTGATTCTCGGCGG
rs62288111_C_EMSA	CCGCCGAGAATCAACACTTGCATGCCGCC	GGCGGCATGCAAGTGTTGATTCTCGGCGG
rs11716984_A_EMSA	CCGCCGGAAACACAAATATGTGGGCCGCC	GGCGGCCCACATATTTGTGTTTCCGGCGG
rs11716984_T_EMSA	CCGCCGGAAACACATATATGTGGGCCGCC	GGCGGCCCACATATATGTGTTTCCGGCGG
rs7957274_T_EMSA	CCGCCGATAGTAAATATTAATTTGCCGCC	GGCGGCAAATTAATATTTACTATCGGCGG
rs7957274_C_EMSA	CCGCCGATAGTAAACATTAATTTGCCGCC	GGCGGCAAATTAATGTTTACTATCGGCGG
rs2858870_A_EMSA	CCGCCGAAGAAAATATTTTCCTTCGCCGCC	GGCGGCGAAGGAAAATATTTTCTTCGGCGG
rs2858870_G_EMSA	CCGCCGAAGAAAATGTTTTCCTTCGCCGCC	GGCGGCGAAGGAAAACATTTTCTTCGGCGG
ESR1_monomer	CCGCCGTATCTCTAAA**TAC**TATTTTCTTTCAGCCGCC	GGCGGCTGAAAGAAAATAGTATTTAGAGATACGGCGG
PVT1_monomer	CCGCCGACTTGGTAAA**TAC**TATTTACTGAATGCCGCC	GGCGGCATTCAGTAAATAGTATTTACCAAGTCGGCGG
QSOX1_monomer	CCGCCGAATCAGCAAA**TAC**TATTTGTTGAGTGCCGCC	GGCGGCACTCAACAAATAGTATTTGCTGATTCGGCGG
KAT6B_monomer	CCGCCGAGGGAACAAA**TAC**TATTTACACTTGGCCGCC	GGCGGCCAAGTGTAAATAGTATTTGTTCCCTCGGCGG
ATP9A_monomer	CCGCCGACTGCGCAAA**TAC**TATTTGCTGAATGCCGCC	GGCGGCATTCAGCAAATAGTATTTGCGCAGTCGGCGG
ESR1_no binding	CCGCCGTATCTCTGAGGAGGTTCTTTCAGCCGCC	GGCGGCTGAAAGAACCTCCTCAGAGATACGGCGG
PVT1_no binding	CCGCCGACTTGGTGAGGAGGTACTGAATGCCGCC	GGCGGCATTCAGTACCTCCTCACCAAGTCGGCGG
QSOX1_no binding	CCGCCGAATCAGCGAGGAGGTGTTGAGTGCCGCC	GGCGGCACTCAACACCTCCTCGCTGATTCGGCGG
KAT6B_no binding	CCGCCGAGGGAACGAGGAGGTACACTTGGCCG	GGCGGCCAAGTGTACCTCCTCGTTCCCTCGGC

	CC	GG
ATP9A_no binding	CCGCCGACTGCGCGAGGAGGTGCTGAATGCCGCC	GGCGGCATTCAGCACCTCCTCGCGCAGTCGGCGG

Accession numbers to ChIP-seq datasets

Name	Accession number	Source
Human_MCF7_FoxA1_ChIP-seq	ENCFF728TJB	ENCODE
Human_T47D_FoxA1_ChIP-seq	ENCFF593ZQY	ENCODE
Human_HepG2_FoxA1_ChIP-seq	ENCFF648VIL	ENCODE
Human_A549_FoxA1_ChIP-seq	ENCFF204MVK	ENCODE
Human_ECC_FoxA1_ChIP-seq	wgEncodeEH001586	ENCODE
Human_T47D_CTCF_ChIP-seq	wgEncodeEH001656	ENCODE
Human_T47D_Jund_ChIP-seq	wgEncodeEH003321	ENCODE
Human_T47D_EP300_ChIP-seq	wgEncodeEH001602	ENCODE
Human_T47D_GATA3_ChIP-seq	wgEncodeEH001639	ENCODE
Human_T47D_DMSO_FoxA1_ChIP-seq	GSM2242433/4	GEO
Human_T47D_BYL719_FoxA1_ChIP-seq	GSM2242428/9	GEO
Human_T47D_DMSO_Input_ChIP-seq	GSM2242430	GEO
Human_T47D_DMSO_ATAC-seq	GSM2241147	GEO
Human_T47D_BYL719_ATAC-seq	GSM2241149	GEO
Human_T47D_BYL_Input_ChIP-seq	GSM2242425	GEO
Human_MCF7_FoxA1_ChIP-seq	GSM798437	GEO*
Human_MCF7_FoxA1_ChIP-seq	GSM986065	GEO*
Human_MCF7_FoxA1_ChIP-seq	GSM1470026	GEO*
Human_MCF7_FoxA1_ChIP-seq	GSM631474	GEO*
Human_MCF7_FoxA1_ChIP-seq	GSM588930	GEO*
Human_MCF7_FoxA1_ChIP-seq	GSM798438	GEO*
Human_MCF7_FoxA1_ChIP-seq	GSM798436	GEO*
Human_T47D_FoxA1_ChIP-seq	GSM631473	GEO*
Human_A549_FoxA1_ChIP-seq	GSM1010826	GEO*
Human_DU145_FoxA1_ChIP-seq	GSM1164146	GEO*
Human_LNCaP_FoxA1_ChIP-seq	GSM699634	GEO*
Human_HepG2_FoxA1_ChIP-seq	GSM803432	GEO*
Human_VCap_FoxA1_ChIP-seq	GSM1463462	GEO*
Human_prostate_FoxA1_ChIP-seq	GSM1716762	GEO*
Human_LNCap_FoxA1_ChIP-seq	GSM1576451	GEO*
Human_LNCap_FoxA1_ChIP-seq	GSM1068136	GEO*
Human_MCF7_FoxA1_ChIP-seq	GSM631474	GEO*

* data collected from Cistrome DB (http://cistrome.org/db/#/)

Cooperativity values (Omega)

Motif	Omega values
D0	56.3 ± 11.9
D2	0.26 ± 0.02
C0	1.8 ± 1.3
C1	0.005 ± 0.004
C-1	0.1 ± 0.06
DIV0	56.3 ± 11.9
DIV1	0.3 ± 0.2
DIV2	0.26 ± 0.02
DIV3	NA
DIV4	0.97 ± 0.34
DIV5	1.15 ± 0.7
DIV6	1.06 ± 0.12
DIV7	1.7 ± 0.27
DIV8	1.83 ± 0.37
DIV9	1.67 ± 0.23
DIV10	1.86 ± 1.56
ESR1_EMSA	41 ± 5.6
ATP9A_EMSA	26.5 ± 3.4
KAT6B_EMSA	9.24 ± 0.53
PVT1_EMSA	60.7 ± 25.12
QSOX1_EMSA	45.3 ± 5.9
rs2104047_A	78.3 ± 17.9
rs2104047_G	0.4 ± 0.02
rs2941742_A	102.7 ± 8.9
rs2941742_G	5 ± 0.5
rs281038_A	22 ± 1.8
rs281038_G	0.25 ± 0.03
rs6990531_T	19.4 ± 2.8
rs6990531_G	9.9 ± 2
rs7957274_T	8.5 ± 1.3
rs7957274_C	0.27 ± 0.13
rs34466261_-	10.9 ± 3.2
rs34466261_C	6.5 ± 1.2
rs2149943_T	49.3 ± 3.5
rs2149943_C	2.5 ± 0.6
rs7007731_T	1.8 ± 0.1
rs7007731_C	0.6 ± 0.2

Position weight matrices

FOXA1 monomer:

A	C	G	T
0.498	0.005	0.001	0.496
0.681	0.037	0.248	0.034
0.596	0.001	0.096	0.307
0.114	0.001	0.884	0.001
0.001	0.153	0.001	0.845
0.931	0.067	0.001	0.001
0.985	0.013	0.001	0.001
0.997	0.001	0.001	0.001
0.001	0.714	0.001	0.284
0.997	0.001	0.001	0.001

FOXA1 DIV:

A	C	G	T
0.408083	0.238106	0.104147	0.249663
0.370568	0.126751	0.04029	0.462392
0.508194	0.155604	0.215155	0.121047
0.550038	0.057125	0.143032	0.249805
0.535629	0.105077	0.334975	0.02432
0.144324	0.317983	0.046022	0.491671
0.887573	0.096705	0.004179	0.011543
0.907495	0.078146	0.002895	0.011465
0.922819	0.005392	0.00476	0.067029
0.001674	0.085943	0.000433	0.91195
0.853512	0.009705	0.124736	0.012046
0.004661	0.012344	0.00354	0.979454
0.005441	0.006236	0.02498	0.963343
0.021645	0.023376	0.131625	0.823354
0.447224	0.011819	0.377024	0.163933
0.078756	0.382721	0.103204	0.435319
0.197284	0.168026	0.059636	0.575053
0.154022	0.225591	0.101175	0.519212
0.417846	0.036054	0.093392	0.452708
0.220348	0.124388	0.292585	0.362679
0.125403	0.166849	0.23204	0.475708
0.252423	0.213793	0.133661	0.400123

FOXA1 CON:

A	C	G	T
0.255988	0.187838	0.168539	0.387635
0.217424	0.145777	0.126323	0.510477
0.248268	0.247286	0.16156	0.342885
0.379054	0.132957	0.083227	0.404763
0.259986	0.186218	0.129803	0.423993
0.011097	0.05402	0.006238	0.928645
0.467502	0.057018	0.3815	0.093979

0.010907	0.023521	0.006686	0.958886
0.009546	0.019678	0.044267	0.926509
0.038667	0.039718	0.226901	0.694713
0.567753	0.025968	0.335131	0.071148
0.079471	0.555019	0.076438	0.289072
0.25199	0.088207	0.009753	0.65005
0.327687	0.172313	0.172313	0.327687
0.65005	0.009753	0.088207	0.25199
0.289072	0.076438	0.555019	0.079471
0.071148	0.335131	0.025968	0.567753
0.694713	0.226901	0.039718	0.038667
0.926509	0.044267	0.019678	0.009546
0.958886	0.006686	0.023521	0.010907
0.093979	0.3815	0.057018	0.467502
0.928645	0.006238	0.05402	0.011097
0.423993	0.129803	0.186218	0.259986
0.404763	0.083227	0.132957	0.379054
0.342885	0.16156	0.247286	0.248268
0.510477	0.126323	0.145777	0.217424
0.387635	0.168539	0.187838	0.255988
